# Coupled Multivariate Analyses Reveal Separate Climate and Local Drivers of Temporal and Spatial Change in a Coastal Marine Ecosystem

**DOI:** 10.1002/ece3.71637

**Published:** 2025-06-27

**Authors:** Tyler R. Abruzzo, Michael G. Frisk, Liam Butler, Matthew Sclafani, Paul Nunnenkamp, Rachel Sysak, Robert M. Cerrato

**Affiliations:** ^1^ School of Marine and Atmospheric Sciences Stony Brook University Stony Brook New York USA; ^2^ Cornell Cooperative Extension of Suffolk County Riverhead New York USA; ^3^ Marine Resources Division New York State Department of Environmental Conservation Kings Park New York USA

**Keywords:** local and climate drivers, monitoring, multivariate analyses, nekton, temporal and spatial community structure

## Abstract

Extensive temporal and spatial monitoring data provide an opportunity to identify the drivers of ecosystem change and to understand spatial relationships useful to conservation and management. Such data can potentially overcome the considerable intrinsic variability present in sampling and justify the cost of sustained monitoring. In this study, the temporal and spatial structure and trends in the mobile invertebrate and fish assemblage of the Peconic Estuary were identified. Data were obtained primarily from a small mesh trawl survey conducted by the New York State Department of Environmental Conservation from 1987–2020 at 76 locations distributed throughout the system, supplemented by chlorophyll data and regional climate indices. A set of multivariate statistical tools, including K‐means cluster analysis, redundancy analysis, and multiscale ordination, were applied to the data set in a complementary way. Distinctly different drivers for temporal and spatial patterns were found. Abrupt community shifts on a decadal time scale occurred, including a regime shift in 1999–2000, and were driven by changes in regional climate factors as indexed by the unlagged and lagged Atlantic Multidecadal Oscillation and North Atlantic Oscillation. Spatially distinct habitats and assemblages were identified, separating eastern, inshore, and offshore regions of the system. These were differentiated by local conditions in bottom salinity, water depth and depth gradient, DO percent saturation, and water transparency. Each of these regions responded to the climate drivers in a similar way. Notably, annual bottom temperature and chlorophyll *a* were never found to be effective in explaining community variation. Overall, the results of this study suggest that, given the time lags in response, climate‐induced changes in the system can be anticipated by continued monitoring and that conservation and management actions can be applied system‐wide and not restricted to specific areas.

## Graphical abstract

A set of readily available multivariate tools was used in a complementary way to identify and better understand both the inter‐annual and spatial patterns in a fish and mobile invertebrate community in the Peconic Estuary. We found distinctly different drivers for temporal and spatial patterns, with temporal factors operating on a regional scale and spatial factors tied to local sampling conditions. Our findings have important implications suggesting that climate‐induced changes in a system can be anticipated by continued monitoring and that conservation and management actions can be applied system‐wide and not restricted to specific areas.

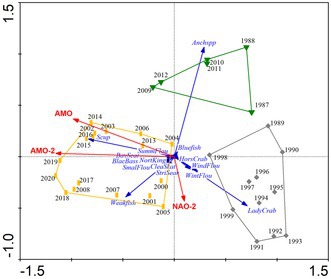

## 1 Introduction

Long‐term and extensive monitoring series of survey data provide an opportunity to understand ecosystem structure and phenomena that would otherwise be unnoticed or at best unexplained (Nygård et al. 2016). To be effective, monitoring surveys must account for the considerable intrinsic variability in sampling to identify patterns in the data. As an example of the magnitude of this variability, Flanagan et al. (2018) found that 36%–59% of the total variation in benthic faunal sampling was due to patchiness, i.e., variation at a scale below the sampling interval. This small‐scale heterogeneity is due to factors such as sampling error, measurement error, and fine‐scale heterogeneity from biotic, abiotic, and anthropogenic processes (Legendre and Legendre 2012). Detecting change may require decades of data, dozens of measurements, and accessory data on potential drivers (Hewitt and Thrush 2019), justifying the value of sustained monitoring.

**FIGURE 1:**
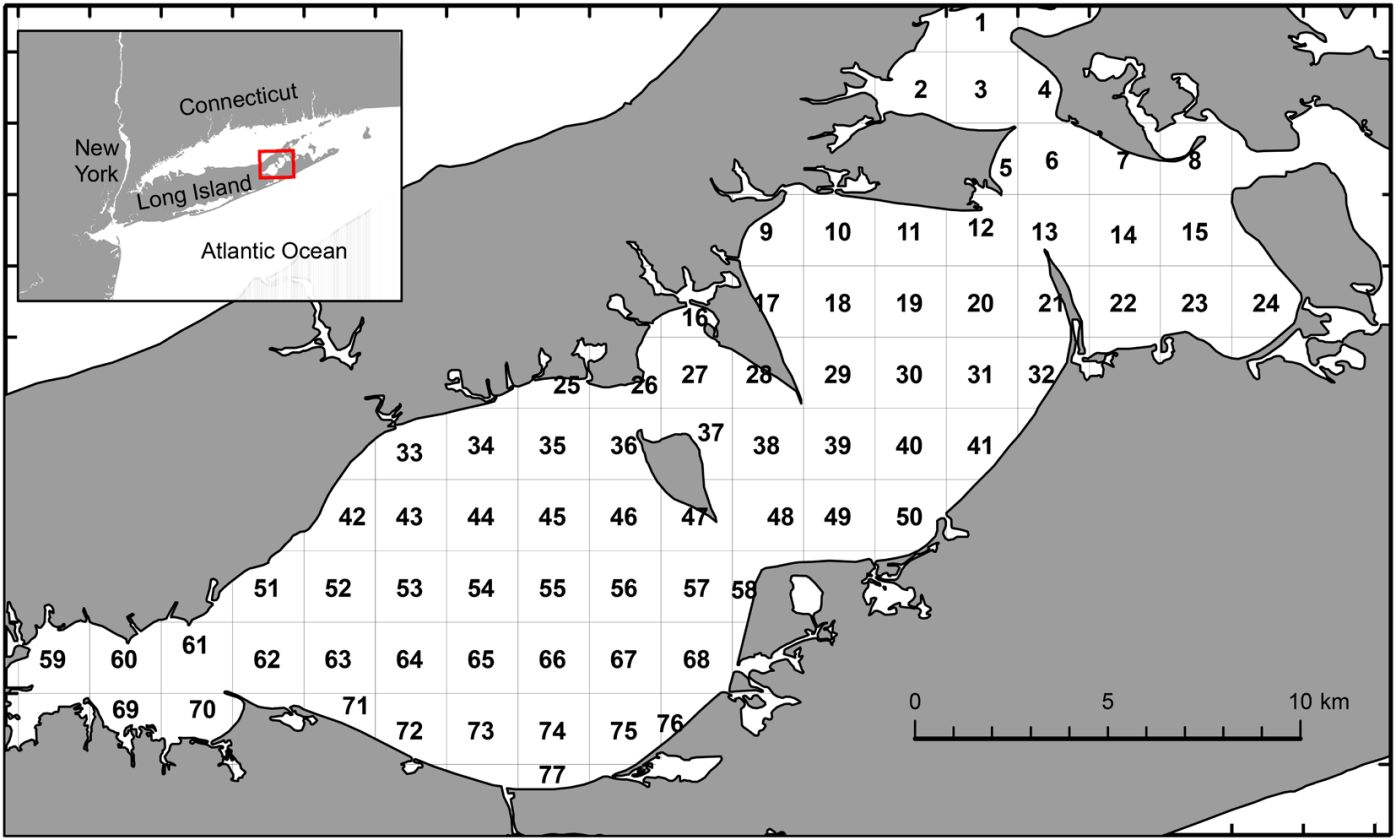
Sampling grid units in the Peconic Estuary, Long Island, New York, USA. Grid unit 59 was removed from data analysis because of low sampling frequency.

The Peconic Estuary, located between the North and South forks of Long Island, New York, USA (Figure 1), was recognized as an Estuary of National Significance in 1993 (Peconic Estuary Partnership 2020). The estuary is temperate in nature and displays a high degree of seasonality. Some species of finfish permanently reside in the estuary, but many species undertake, sometimes long, migrations to use the estuary as a spawning, nursery, or feeding ground (Perlmutter et al. 1956, Buckel et al. 1999, Thorrold et al. 2001). The depth across the estuary ranges from approximately 1.5 m in the west (Flanders Bay) of the estuary to approximately 29 m towards the eastern region (Shelter Island Sound) (Hardy 1976). The faunal and floral assemblages across the estuary are very diverse and provide habitat for a large number of endangered and/or locally protected species (Hornstein 2020). The different regions of the estuary (deep water, shallow water, intertidal), diverse sediment types, and the presence of seagrasses (most notably eelgrass *Zostera marina* ) increase species richness of local and migratory fish and invertebrate species and also provide an optimal habitat for spawning and nursery grounds (Hardy 1976; Peconic Estuary Partnership 2020). The sediment type found across the estuary varies from sandy or gravelly sand bottom to a more clayey silt and/or silty clay bottom (Coch 2019; Cerrato and Maher 2007; Cerrato et al. 2009, 2010; Katuna 1974).

Beginning in 1987, the New York State Department of Environmental Conservation (NYSDEC) has conducted a small mesh trawl survey for finfish and mobile invertebrates in the Peconic Estuary. This monitoring study not only collects faunal data but also tow‐specific bottom temperature, salinity, dissolved oxygen, water depth, and Secchi depth. The survey collects on average about 366 trawl samples every year during the period from late April to early November. Coupled with other monitoring data available for the Peconics system (e.g., chlorophyll *a*) and regional climate indices (e.g., Atlantic Multidecadel Oscillation index), the combined data set provides a rare opportunity to characterize the structure and scale of variation, both temporally and spatially, in an important ecosystem.

The aim of this research was to assess the temporal and spatial structure of the Peconic Estuary community of finfish and mobile invertebrates during the period 1987 to 2020 and to identify the principal environmental drivers that likely play a critical role in creating and maintaining this structure. A complementary suite of multivariate statistical techniques centered on redundancy analysis (RDA) was used. Data analysis in this study was designed to take advantage of the extensive monitoring data set to identify whether changes in community structure have occurred over time, whether changes have been gradual or abrupt, whether they have occurred throughout the system or in restricted areas, and whether the principal drivers of community structure operate at a local or regional scale. Characterizing the structure and scale of variation in the fish and mobile invertebrate community and its relationship with the environment has obvious consequences to conservation, resource management, and ecosystem services, as the estuary is an important breeding ground for many fish and invertebrate species and is already threatened by anthropogenic environmental degradation (Abruzzo 2015; Hardy 1976; Lewis et al. 1997).

## 2 Materials and Methods

### 2.1 Survey and Data Collection

The New York State Department of Environmental Conservation (NYSDEC) has conducted a fishery‐independent small mesh trawl survey for finfish and mobile invertebrates in the Peconic Estuary System since 1987. The survey area consists of Flanders Bay, Great Peconic Bay, Little Peconic Bay, and several smaller bays (Southold Bay, Noyack Bay, and Shelter Island Sound) bordering Shelter Island but not including the North and South channels around Shelter Island. The allocation of stations is based on 77 1‐min latitude and 1‐min longitude sampling grid units (~1.85 × 1.41 km) (Figure 1). Each week from May through October, the survey targeted 16 randomly chosen grid units, with annual start dates beginning late April and some end dates spilling over into early November. Exceptions to this design plan included no trawls after mid‐July in 2005, none until mid‐July in 2006, none before August in 2008, none in May 2010, and none before late June 2020. Between 1987 and 2020, a total of 12,461 samples were collected.

Samples were collected using a 4.9 m semi‐balloon otter trawl with a 2.5 cm mesh body and a 1 cm codend liner. The trawl net has a 5.2 m head rope and 6.4 m bottom rope with small floats, as well as a 0.5 cm tickler chain attached to the bottom rope (Nunnenkamp 2020; Weber et al. 1998). Tows were set for 10 min at a target speed of 2.5 knots (Nunnenkamp 2020). Tows started from the approximate center of each grid unit and were towed towards a random location in a straight line. At the start of each tow, bottom temperature (°C), salinity (ppt, practical salinity units), dissolved oxygen (mg/L), depth (m) and Secchi depth (m) were recorded. Depth was also recorded at the end of the tow, allowing the calculation of the change in depth during a tow. All species contained in the tow were identified and counted. Grid unit 59 was removed from the survey because of problems collecting samples at that location. Overall, every remaining grid unit was trawled an average of 4.8 (±0.52 SD) times per year, although 21 grid units had 1–2 years with no sampling and 4 grid units had 3–5 years of missing data.

Environmental data from the trawl survey was supplemented with climatic indices and Peconic Estuary data from the Suffolk County Department of Health Services. Trawl data are available at data.ny.gov (data.ny.gov/d/abww‐z5t5 and /d/9if6‐dz5v). The Atlantic Multidecadal Oscillation Index (AMO) and the North Atlantic Oscillation Index (NAO) were obtained from National Oceanic and Atmospheric Administration websites (psl.noaa.gov/data/timeseries/AMO/ and www.ncdc.noaa.gov/teleconnections/nao/). Chlorophyll data from a set of water quality monitoring stations was obtained from the Suffolk County Department of Health Services, Office of Ecology at the following link: gis.suffolkcountyny.gov/portal/home/item.html?id=5d4b53ec44204219a8da685f1859e096.

### 2.2 Data Processing

Catch per unit effort (CPUE) data were trimmed to remove rare species. In this study, a species was considered rare and was deleted if it was caught less than a total of 10 times over the course of the survey. This criterion addresses the caution by Poos and Jackson (2012) concerning the impact of rare species, since so few occurrences of a species over such a large number of trawl samples would not impact ecological patterns in the multivariate analyses. A total of 54 rare species were removed, resulting in a total of 69 species retained in the data set. In addition, some species were combined into their respective broader functional groups because of the uncertainty in making consistently accurate identifications. These included spider crabs (*Libinia* spp.), herrings (*Clupeidae* spp.), anchovies (*Anchoa* spp.), and squid (*Cephalopoda* spp.). Annual CPUE was calculated on species and species groups prior to any further processing.

Annual average CPUE data were Hellinger transformed (Legendre and Gallagher 2001) prior to multivariate analysis. This transformation is particularly useful for data sets with many 0 counts, and it down‐weights highly abundant species, preventing them from dominating the analysis. In addition, when combined with Euclidean distance, the distance metric utilized by all the multivariate analyses in this study, this transformation provides an intuitive and ecologically reasonable measure of compositional dissimilarities in community structure (Legendre and Gallagher 2001). A preliminary detrended correspondence analysis on the CPUE data confirmed that the gradients along each axis were below 2.5 SD (standard deviation of species turnover), allowing linear rather than unimodal multivariate methods (ter Braak and Smilauer 2002).

Environmental data used in the multivariate analyses were either calculated as annual averages or derived from the available data. Annual averages for the trawls in each grid unit were calculated from bottom temperature (Temp;°C), bottom salinity (Sal; ppt), bottom DO (DO; mg/L), DO percent saturation (DOSat; %), depth (Depth; m), and Secchi depth (Secchi; m) recorded at the start of each tow. Change in depth (delDepth; m) was taken as the average of the difference in the tow start and end depths. Measures derived from the data also included the percent of measurements during the year with DO < 3 mL/L (PerHyp), with DO saturation > 115% (PerGrSat), and that were in the highest (PerHighT) or lowest (PerLowT) 10% of Temp measurements for all years. Total chlorophyll *a* (Chla; μg/L) was estimated for each trawl from the nearest available water quality sample from the Suffolk County Department of Health Services monitoring program and calculated as an annual average of the trawls in the grid unit. The average annual AMO index was calculated using all months since it is already a large scale, temporally averaged time series (Enfield et al. 2001; Trenberth and Shea 2006). The average annual winter (January, February, March) NAO index was used since atmospheric variability over the North Atlantic is most prominent during this time period (Hurrell et al. 2003). Since marine ecosystems might not instantaneously respond to large‐scale environmental variables (Báez et al. 2021), 1‐ and 2‐year lags for bottom Temp (BT‐1, BT‐2), NAO (NAO‐1, NAO‐2), and AMO (AMO‐1, AMO‐2) were also included in the environmental data set. The forward selection process described in the next section can distinguish which, if any, of these time lags explain the largest amount of faunal variation.

### 2.3 Statistical Methods

To capture the temporal and spatial patterns in the finfish and mobile invertebrate community, the data were analyzed using a complementary set of statistical techniques. K‐means cluster analysis was used to determine whether temporal or spatial groups with similar community characteristics could be identified in the data set. Redundancy analysis (RDA) was the principal means of identifying the environmental drivers of community structure. Multiscale ordination (MSO) was used as a diagnostic tool to evaluate scale dependence in the identified biotic‐environmental RDA relationship, to indicate the possibility of missing environmental drivers, to measure the stability of residuals in the identified biotic‐environmental relationship, and to assess the presence of temporal or spatial autocorrelation in the residuals. A univariate Spearman's rank order correlation was also performed on the mean annual CPUE of species vs. year to assess trends in catch across the 34‐year survey period.

#### 2.3.1 K‐Means Cluster Analysis

K‐means cluster analysis (Legendre and Legendre 1998) was carried out on the Euclidean distance matrix derived from Hellinger‐transformed CPUE data. K‐means is non‐hierarchical and finds a range of groups from 2 to *r*, where *r* is an arbitrarily large number of groups. The CH index (Calinski and Harabasz 1974), a multivariate *F*‐statistic that compares the between‐cluster sum‐of‐squares to the within‐cluster sum‐of‐squares, was used to evaluate each group solution and suggest the best partitioning of the data. K‐means cluster analysis was carried out using the R library vegan (Oksanen et al. 2022). Because the CH index undervalues unequal size partitions (Borcard et al. 2011), Oksanen et al. (2022) recommends considering several groups near the maximum, and not just the one corresponding to the maximum, when groups are unequal in size.

#### 2.3.2 Redundancy Analysis (RDA)

RDA is a multivariate direct gradient analysis technique that explicitly combines ordination of samples based on species catch data with regression on the environmental data (Jongman et al. 1995). A forward selection process was used to identify environmental variables to include in the regression model (Jongman et al. 1995). At each step in the process, the environmental variable explaining the largest amount of faunal variation was selected and its effect removed before the next best fitting variable was considered. This process of adding environmental variables was continued until the model with the smallest Akaike Information Criterion (AICc) was identified (Burnham and Anderson 2002; Hastie et al. 2009). RDA analyses were conducted using the software Canoco 4.5 (Microcomputer Power, Ithaca NY) (ter Braak and Smilauer 2002) and the vegan package of R (R Foundation for Statistical Computing, Vienna, Austria).

To examine whether temporal changes in community structure were gradual or abrupt, and persistent, a change point analysis was conducted with the mcp() function in the MCP library (Lindeløv 2020). An uninformative default prior was used in the Bayesian regression analysis, and the model was constructed as a change point between two levels. This analysis was carried out with sample scores along the first RDA axis vs. year. Rerunning the analysis using comparable scores from first principal component analysis resulted in essentially an identical outcome and is not reported here.

#### 2.3.3 Empirical Variograms and Multiscale Ordination (MSO)

The temporal and spatial structure of the species assemblage was examined by constructing an empirical variogram of the multivariate faunal data for each site (Wagner 2003):

(1) $$\gamma \left(h\right)=\sum_{i=1}^{p}\frac{1}{2n_{h}}\sum_{a,b|h_{\mathrm{ab}}\approx h}{\left(x_{\mathrm{ia}}-x_{\mathrm{ib}}\right)}^{2}$$

where $\gamma \left(h\right)$ is the empirical semivariance of the faunal data at time interval or distance interval h, $x_{\mathrm{ia}}$ and $x_{\mathrm{ib}}$ are Hellinger transformed CPUE values for species i (i=1top) in samples a and b, respectively, and the inner summation is over all pairs of samples separated by a time interval or geographic distance of approximately h. It should be noted that applying equation (1) to all pairs of samples, instead of a temporal or distance interval subset, yields $s^{2}$, the total sample variance (Bachmaier and Backes 2008).

Interval increment sizes were chosen for interpretability with 1‐year increments for temporal and 2000 m, the approximate average distance to nearest neighbor grid units, for spatial. Semivariances at time or spatial intervals beyond half the maximum were not interpreted since not all samples can be used in the variance calculation (Wagner 2003, 2004). Specifically, samples in the center of a time series or study area cannot be paired with other samples when intervals are larger than half the maximum. This creates a bias that gets progressively worse as the interval size increases.

Multiscale ordination (MSO) extended the temporal and spatial structure analysis to examining biotic‐environmental relationships by inserting RDA regression results into the variogram (Wagner 2003, 2004, Wagner and Fortin 2005). It does this by partitioning the $x_{\mathrm{ia}}$ and $x_{\mathrm{ib}}$ pairs in Equation (1) into fitted and residual parts $\left({\hat{x}}_{\text{iafit}}+{\hat{x}}_{\text{iares}}\right)$ and $\left({\hat{x}}_{\text{ibfit}}+{\hat{x}}_{\text{ibres}}\right)$, respectively. Substituting these into Equation (1) leads to:

(2) $$\gamma \left(h\right)=\gamma_{\mathrm{fit}}\left(h\right)+\gamma_{\mathrm{res}}\left(h\right)+\gamma_{\text{cross}}\left(h\right)$$

The first two terms on the right‐hand side are variograms of the fitted and residual values. The third term is twice the covariance between the fitted and residual differences for distance class h (Wagner 2003). Multivariate regression estimates of species‐environmental relationships were obtained from the forward selection, minimum AICc RDA solution, and variograms were created using the rda(), mso(), and msoplot() functions in the vegan package of R (R Foundation for Statistical Computing, Vienna, Australia). The code for mso() and msoplot() was created and first published by Wagner (2004).

MSO provided diagnostic tools for the RDA results to evaluate scale dependence in the biotic‐environmental relationship, stationarity of the residuals, and spatial autocorrelation in the residuals using methods in Wagner (2003). Scale dependence in the biotic‐environmental relationship was tested by constructing a Bonferroni corrected point confidence interval around $\gamma \left(h\right)$ and determining if the sum of the variograms $\gamma_{\mathrm{fit}}\left(h\right)+\gamma_{\mathrm{res}}\left(h\right)$ lies wholly within it. If not, the species‐environmental relationship is scale dependent. Stationarity of the residuals was examined by determining whether $\gamma_{\mathrm{res}}\left(h\right)$ reached a sill or stable level after short time or distance intervals and remained there over most of the interval range. Presence of a trend in $\gamma_{\mathrm{res}}\left(h\right)$ would indicate that important environmental variables were missing in the RDA model. Spatial autocorrelation in the residuals was tested by a series of Bonferroni adjusted Mantel tests (Mantel 1967) between a Euclidean distance matrix formed by the residuals within a class interval and a temporal or geographic matrix for that interval. A significant outcome for a class interval would indicate that small scale autocorrelation due to biotic processes (Legendre 1993, Wagner 2004) was present at that interval class. The scale dependence and spatial autocorrelation analyses are built into the msoplot() function in the vegan package of R (R Foundation for Statistical Computing, Vienna, Australia).

#### 2.3.4 Multivariate Approach

The multivariate techniques were applied to the spatial–temporal data set in stages. Data analysis initially focused on assessing separately the spatially averaged temporal structure and the temporally averaged spatial structure in the data, and in identifying the environmental variables responsible for patterns in community structure. Building on these initial results, more spatial detail was added to evaluate the full assemblage complexity in the Peconics data set. In all analyses, CPUE data were spatially or temporally averaged prior to applying the Hellinger transformation.

## 3 Results

### 3.1 Spatially Averaged Temporal Variability

Temporal analysis identified three coherent species assemblages related to changes in regional environmental factors. Examining annual variability after averaging across all grid units, the maximum CH index solution suggested by K‐means cluster analysis separated the survey years into two distinct time periods (1987–1999 and > 2000), with only two exceptions (Figure S1). The two exceptions were the years 2010 and 2011, which were included with the 1987–1999 group. The next largest CH index solution separated out 1987–1988 and 2009–2012 into a third assemblage group. This solution, because unequal size groups are undervalued (Borcard et al. 2011), was retained for further consideration as suggested by Oksanen et al. (2022). Subsequent RDA analyses confirmed it was clearly associated with environmental variation, so three groups were retained and designated as pre2K (1989–1999), pst2K (2000–2020 minus 2009–2012), and trans (i.e., transitory, 1987–88 and 2009–2012).

**FIGURE 2:**
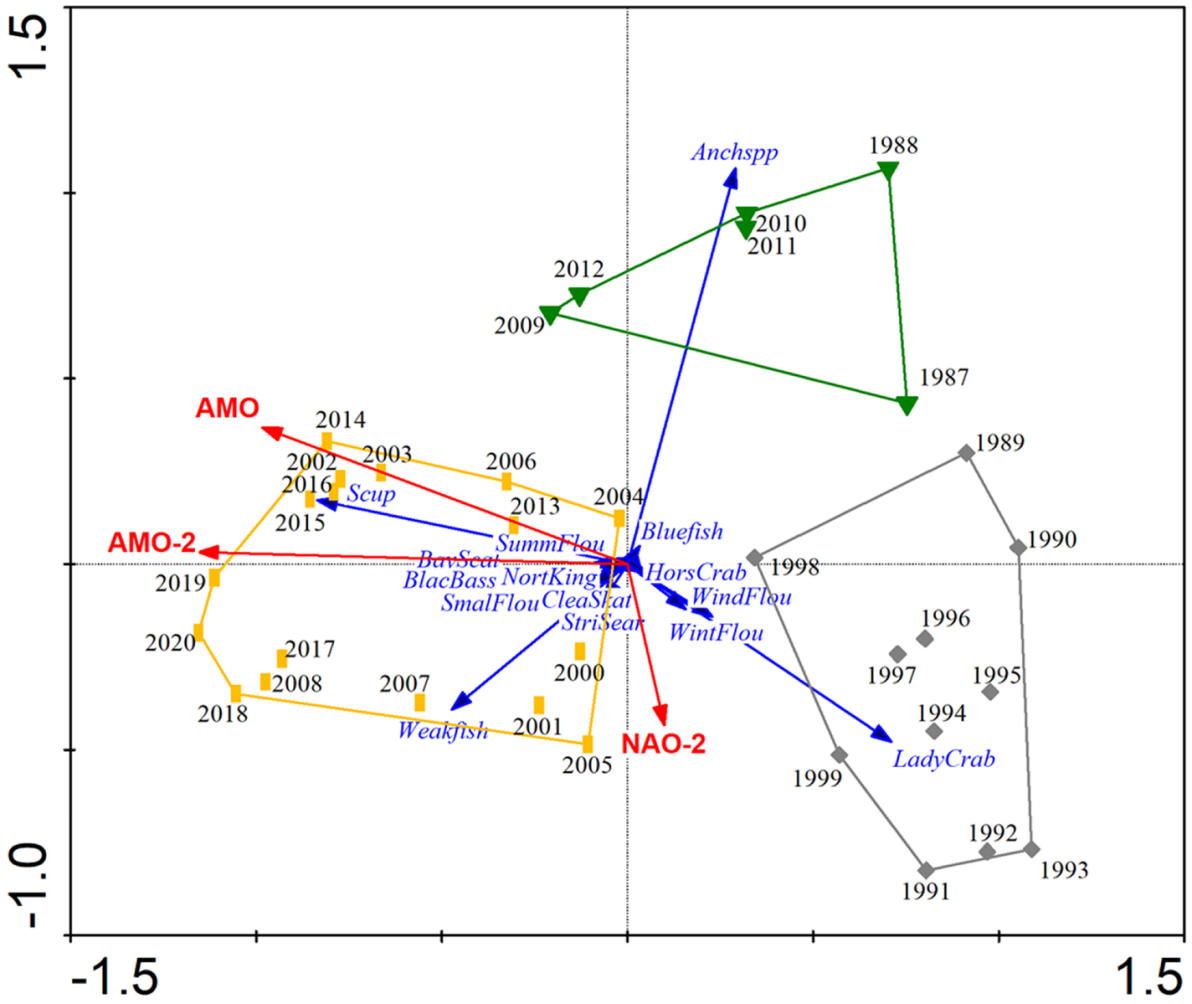
Redundancy Analysis (RDA) of spatially averaged temporal patterns in community structure. The first two RDA axes are plotted. The points are annual community structure scores derived from Hellinger transformed average CPUE species scores. Envelopes drawn around points designate groups identified in the K‐means cluster analysis. The blue arrows represent species and the red arrows represent environmental variables. The arrows characterize the direction of steepest increase for the species or quantitative environmental variable. The origin is the mean of the variable and decreasing values extend through the origin in the direction opposite the head of the arrow. Only the environmental variables that were included in the minimum AICc model are plotted. The number of species plotted was reduced for clarity.

Redundancy analysis (RDA) resulted in a minimum AICc model with three environmental variables: the annual AMO lagged by 2 years (AMO‐2), the average winter NAO lagged by 2 years (NAO‐2), and the annual AMO (Figure 2, Table 1). These three variables explained 45.7% of the total Hellinger transformed community variation. All selected environmental variables were climatic indices, and notably absent from the forward selection results were any local environmental variables such as bottom temperature, chlorophyll *a*, or dissolved oxygen. Excluding all AMO and NAO variables in RDA analysis resulted in selecting BT‐1, BT‐2, and Tchl, but the explained variance was only about half as much as when the climatic variables were included (24.5% vs. 45.7%).

**TABLE 1:** RDA forward selection results, eigenvalues, and AICc for each temporal, spatial, and combined analysis.

Analysis	Variable selected	Eigenvalue	∑ Eigenvalues	AICc
Spatially averaged temporal variability	AMO‐2	0.262	0.262	−93.766
	NAO‐2	0.099	0.361	−96.084
	AMO	0.096	0.457	**−98.856**
	PerHyp	0.036	0.493	−98.220
Temporally averaged spatial variability	Sal	0.197	0.197	−266.062
	DoSat	0.194	0.391	−284.849
	Depth	0.057	0.448	−290.023
	delDepth	0.038	0.486	−293.084
	Secchi	0.022	0.508	**−293.979**
	Chla	0.010	0.518	−293.037
*Temporal changes in geographic regions*				
Eastern	AMO‐2	0.312	0.312	−97.970
	NAO‐2	0.072	0.384	−99.149
	AMO	0.075	0.459	−100.800
	Secchi	0.049	0.508	**−101.060**
	PerHyp	0.043	0.551	−100.973
Inshore	delDepth	0.302	0.302	−94.047
	AMO‐2	0.101	0.403	−96.782
	NAO‐2	0.077	0.480	−98.714
	AMO	0.046	0.526	−98.895
	PerHyp	0.053	0.579	**−99.729**
	Depth	0.035	0.614	−99.228
Offshore	AMO‐2	0.238	0.238	−91.560
	NAO‐2	0.115	0.353	−94.543
	AMO	0.094	0.447	−97.117
	Depth	0.049	0.496	**−97.303**
	PerHighT	0.029	0.525	−96.121

*Note:* Minimum AICc values are indicated in bold. Environmental variables are listed in order of selection. Eigenvalues represent the fraction of variance explained.

The AMO‐2 and AMO gradient is aligned with the first RDA axis and indicated that the clear temporal separation in community structure found in the K‐means analysis was associated with below average AMO‐2, AMO values during 1987–1999 and above average AMO‐2, AMO values for 2000–2020 with the exception of 2010–2011. Bayesian change point regression analysis of the annual community structure scores along the first RDA axis vs. year confirmed two distinct levels in community structure with a shift between 1999–2000 ($\bar{x}$ = 1999.6) and a 95% credible interval of (1998.5, 2001) (Figure S2). No overlap in the mean RDA scores' credible intervals was found between 1987 and 999 ($\bar{x}$ = 0.80, 95% credible interval of 0.57–1.02) and 2000–2020 ($\bar{x}$ = −0.50, 95% credible interval of −0.68 to −0.32). This community structure change corresponds to a shift in the AMO from cold to warm phase that occurred in the mid‐ to late‐1990s (Trenberth and Shea 2006). The second RDA axis is primarily associated with NAO‐2 and a small component of AMO orthogonal to the first RDA axis. The third assemblage group suggested by the K‐means analysis (trans) is associated with below average NAO‐2 values and this second RDA axis.

RDA predicted Hellinger transformed annual CPUE fit the observed species data very well (Figure S3). The fit suggests that the model created from the three climatic variables was reasonable in describing the trends seen for individual species. Notable changes apparent in the RDA triplot (Figure 2) are species that declined after the 1990s (e.g., Winter Flounder, Lady Crab, Horseshoe Crab, Windowpane Flounder, Fourbeard Rockling, and American Sand Lance), those that increased in the 2000s (Bay Scallop, Clearnose Skate, Northern Kingfish, Smallmouth Flounder, Black Sea Bass, Scup, Summer Flounder, Hogchoker, Spotted Hake, Atlantic Moonfish, Weakfish, and Conger Eel), and at least two taxa (Anchovies and Bluefish) associated with the second RDA axis and the periods 1987–1988 and 2009–2012. Most of these taxa were identified in the Spearman's correlation analysis as having a significant correlation between Hellinger transformed CPUE and year (Figure S4B).

The empirical variance (γ(h) in Equation 1) increased with time interval size, indicating that the community assemblage became increasingly different at larger time intervals (Figure S5). There was no evidence of a sill or flattening of the empirical variogram at large time intervals to suggest a maximum assemblage difference had been reached.

MSO results indicated that the temporal structure in the faunal data was captured by the explanatory variables selected in the RDA and that the residuals contained no trend (Figure S5). The variogram formed from RDA predictions (γfit(h)) had an evident increasing temporal trend that strongly paralleled the shape of the empirical variogram, suggesting that the functional form of the RDA model was not misspecified. Points in the variograms formed by the sum γfit(h) + γres(h) lay within the Bonferroni‐corrected point confidence envelope around γ(h), indicating that there were no problems with scale dependence in the biotic‐environmental relationships. The variogram of γres(h) was flat, signifying that the stationarity assumption was met and no unknown environmental factor(s) was present influencing the temporal structure of the residuals. In addition, Bonferroni‐adjusted Mantel tests between residuals and time intervals were non‐significant, excluding the possibility of autocorrelated residuals.

### 3.2 Temporally Averaged Spatial Variability

**FIGURE 3:**
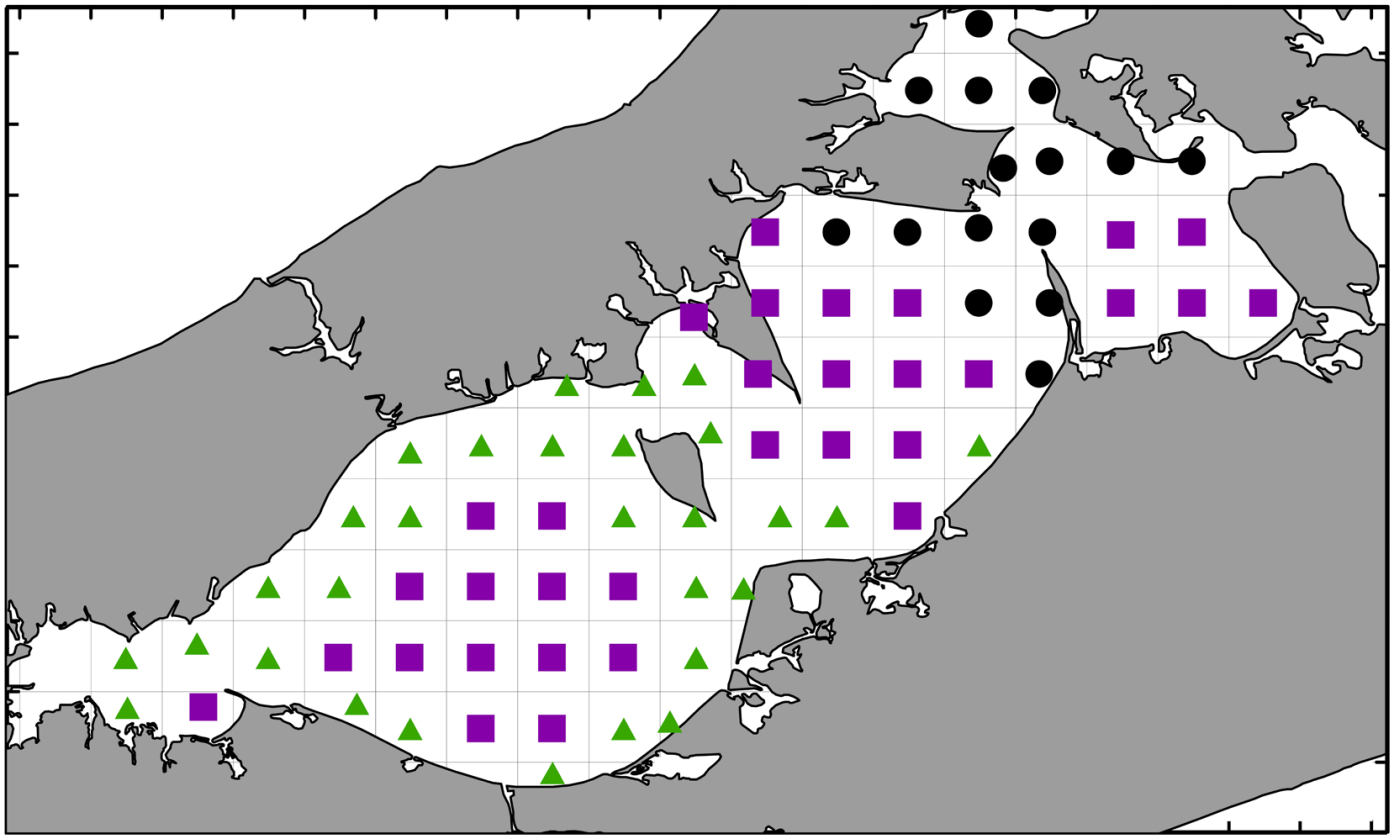
K‐Means cluster analysis of temporally averaged grid unit community structure data. Groups are designated as eastern (black circles), inshore (green triangles), and offshore (purple squares).

Spatial analysis revealed three geographic groups of grid units distinguished by differences in local environmental characteristics. After averaging across years, the CH‐index solution for K‐means cluster analysis separated the grid units into three distinct groups (Figure S6) with a coherent geographic structure (Figure 3). These three regional groups were designated as eastern, inshore, and offshore based on their geographic locations, although this designation has some grid unit exceptions. The CH‐index for two groups was almost identical to the maximum, but it grouped eastern and offshore together. The geographic coherence of the eastern group suggested that it should be considered a separate assemblage.

**FIGURE 4:**
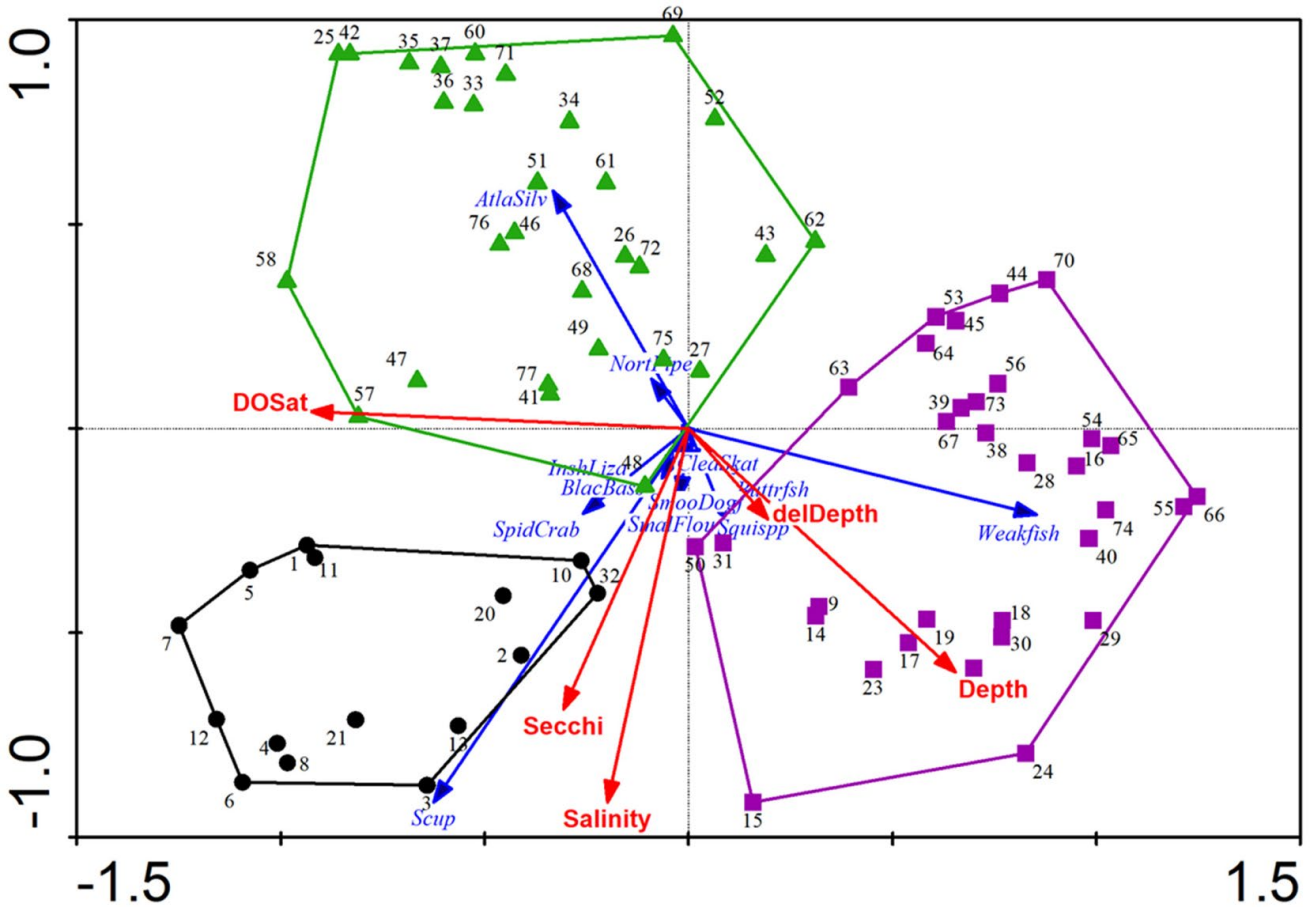
Redundancy Analysis of temporally averaged spatial community structure. The points represent grid unit community structure scores derived from Hellinger transformed average CPUE species scores. Envelopes drawn around points designate grid‐unit groups identified in the K‐means cluster analysis. See Figure 2 caption for further details on interpretation.

Redundancy analysis (RDA) resulted in a minimum AIC model with five environmental variables: bottom salinity (Sal), dissolved oxygen percent saturation (DOSat), starting depth of the trawl (Depth), change in depth during the trawl sample (delDepth), and Secchi depth (Figure 4, Table 1). The five environmental variables explained 50.8% of the total Hellinger transformed community variation, and regional comparisons of these factors are found in Figure S7. Notably absent from the forward selection results was a local temperature variable or chlorophyll *a*. The RDA analysis identified species associated with the three geographic regions. These included Scup, Spider Crab, Black Sea Bass, and Inshore Lizardfish in the eastern assemblage, Atlantic Silverside and Northern Pipefish in the inshore assemblage, and Weakfish, Squid spp., and Butterfish offshore. RDA predicted Hellinger transformed annual CPUE fit the observed grid unit data reasonably well (Figure S8). The predicted vs. observed plots of many species (e.g., SpidCrab, AtlaSilver, BayScal, AnchSpp, Buttrfsh, HorsCrab, Squispp) followed the 1:1 line closely, but tended to underestimate some of the largest observed values. Some species were less well predicted (e.g., AmerLobs, BlackDrum, BlueRunn, FourFlou, HardClam, MoonSnal), but these tended to have 50 or more zero‐grid values across the 76 grid units.

MSO results indicated that the spatial dependence in the faunal data was captured by the explanatory variables selected in the RDA, and that the residuals contained no detectible spatial structure (Figure S9). The variogram formed from RDA predictions γfit(h) had evident spatial structure at all sites and strongly paralleled the shape of the empirical variogram, suggesting that the functional form of the RDA model was not misspecified. Points in the variogram formed by the sum γfit(h) + γres(h) lay within the Bonferroni‐corrected point confidence interval around γ(h), indicating that there were no problems with scale dependence in the biotic‐environmental relationships. The variogram of γres(h) leveled off after the first distance interval (1400 m), and while it fluctuated a small amount (< ±10%), it did not continue to increase with distance class, indicating that the stationarity assumption was met and no unknown environmental factor(s) was present influencing the spatial structure of the residuals. In addition, Bonferroni‐adjusted Mantel tests between residuals and a geographic distance matrix at each distance class interval were non‐significant, excluding the possibility of autocorrelated residuals.

### 3.3 Temporal Changes Within Geographic Regions (Eastern, Inshore, Offshore)

**FIGURE 5:**
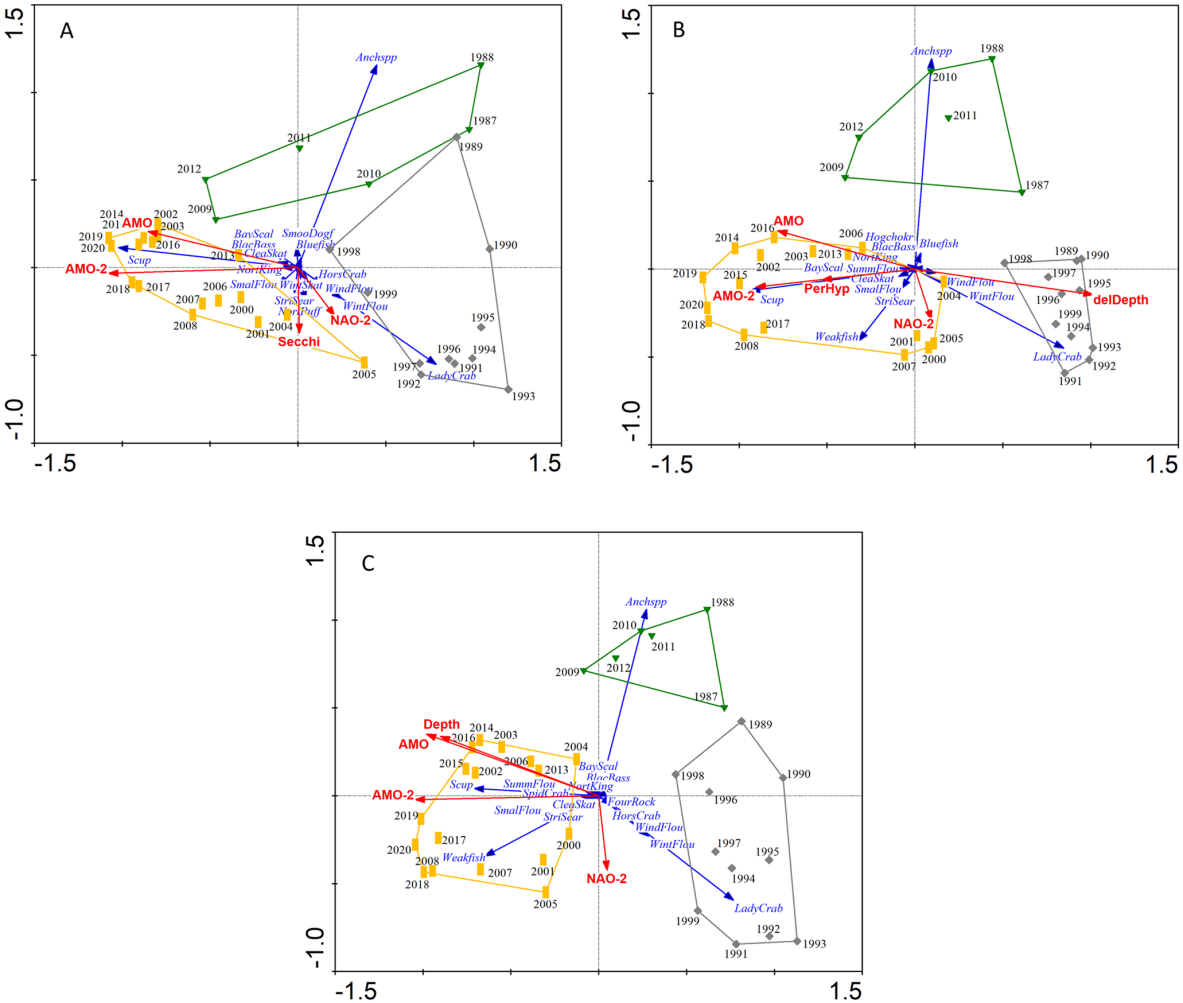
Redundancy Analysis of annual variability for the (A) eastern, (B) inshore, and (C) offshore regional grid‐unit groups. Envelopes and symbols delineate pre2K (1989–1999), trans (1987–1988, 2009–2012), and post2K (2000–2008, 2013–2020) periods. See Figure 2 caption for further details.

Analysis of the temporal changes in each geographic region (eastern, inshore, and offshore) by RDA confirmed that the 1999–2000 regime shift occurred system‐wide and that the same climatic drivers were involved (Figure 5). It also confirmed the separation of the trans periods (1987–1988, 2009–2012) as a separate group associated with the second RDA ordination axis. The AMO‐2 and AMO gradient again dominated the first RDA axis separating pre2K and pst2K years. Bayesian change point analysis of sample scores along the first RDA axis vs. year confirmed two distinct levels in community structure with an estimated shift essentially at the same time in the eastern ($\bar{x}$ = 1999.0, 95% credible interval of 1997.1–2000.0), inshore ($\bar{x}$ = 2000.2, 95% credible interval of 1999.0–2001.8), and offshore ($\bar{x}$ = 1999.5, 95% credible interval of 1999.0–2000.8) regions. As in the case of the full data set, there was no overlap in the mean RDA scores' credible intervals between 1987–1999 and 2000–2020 for any region. A gradient in NAO‐2 separated out the trans years as a group, with no overlap between them and either pre2K or pst2K years (Figure 5). In addition to the climatic drivers, the minimum AICc solution in the forward selection analysis included Secchi depth for the eastern region, the change in depth between the start and end locations of the trawl (delDepth) and the percent of hypoxic events (PerHyp) for the inshore region, and the starting depth of the trawl (Depth) for the offshore region (Table 1). Although some local environmental variables were chosen in the forward selection analysis of the regions, notably absent were common habitat variables like temperature, salinity, or chlorophyll *a*. Explained variation in each regional analysis ranged from 50%–58% (Table 1) and exceeded the explained variance in the spatially averaged temporal analysis (46%) with all three regions combined. MSO results (Figure S10) were similar to the temporal analysis with all regions combined (Figure S5).

### 3.4 Temporal Changes Within Grid Units

Expanding the multivariate analysis to the grid unit scale (34 years × 76 grid units) increased sums of squares variability by two orders of magnitude over previous analyses, decreased the proportion of variance explained by environmental variables in the RDA, but provided no surprises in the explanatory variables selected. Forward selection RDA with all of the environmental variables explained about 31.2% of the total Hellinger transformed community variation. The leading variables selected in order included AMO lagged by 2 years (AMO‐2, 11.9%), starting depth of the trawl (Depth, 4.7%), AMO (3.8%), NAO lagged by 2 years (NAO‐2, 3.8%), bottom salinity (Sal, 1.8%), change in depth during the trawl sample (delDepth, 1.3%), and Secchi depth (Secchi, 1.1%). All other variables added together into the analysis increased the explained variance by less than 3%. RDA using the interactions between the three temporal periods (pre2K, trans, pst2K) and three regional areas (eastern, inshore, and offshore) as categorical variables (i.e., E‐pre2k, E‐tran, E‐pst2K, I‐pre2K, I‐tran, Ipst2K, O‐pre2K, O‐tran, O‐pst2K) explained 38.7% of the Hellinger transformed community variation. Thus, the earlier identified temporal and spatial structure better explained community variability at this level than the original quantitative variables.

## 4 Discussion

The predominant feature characterizing the fish and mobile invertebrate community in the Peconic Estuary was a regime shift that occurred in 1999–2000 and was directly driven by sea surface temperature changes associated with the Atlantic Multidecadal Oscillation. Based on their orientation along the first RDA axis and the Spearman's correlation analysis with year, this shift involved changes in at least 18 taxa, with decreased CPUE in Winter Flounder, Lady Crab, Horseshoe Crab, Windowpane Flounder, Fourbeard Rockling, and American Sand Lance (Figures 2, S3, S4B, Table S2). Increased CPUE was found for Bay Scallop, Clearnose Skate, Northern Kingfish, Smallmouth Flounder, Black Sea Bass, Scup, Summer Flounder, Hogchoker, Spotted Hake, Atlantic Moonfish, Weakfish, and Conger Eel.

While some of the species' changes may be due to a direct effect of temperature on physiological processes, it is likely that most changes were the result of complex population processes and the interplay between migration, spawning, and species interactions. Changes in temperature can induce early or late migratory and spawning behavior and changes in the timing and duration of larvae in different species (Sims et al. 2004; Fincham et al. 2013; Witting et al. 1999; Asch 2015; Slesinger et al. 2021). Since the Peconic Estuary is a temperate system and is extensively used by migratory species, changes in the timing of different species migrations and spawning could have top‐down or bottom‐up effects, resulting in shifting temporal overlaps of predator–prey species. For example, Winter Flounder, Windowpane Flounder, Summer Flounder, and Smallmouth Flounder all changed in CPUE across the regime shift, and they utilize the Bay at different times and for different aspects of their life history. Winter Flounder spawn in estuaries in the late winter to early spring (Fahay 1983; Ziegler et al. 2019). Their eggs and larvae are demersal and present in the early spring. Windowpane Flounder spawn in both estuary and coastal ocean areas from spring to fall, producing two cohorts: (1) a spring‐spawned cohort whose larvae are found in both the estuary and in the coastal ocean and (2) a fall‐spawned cohort whose larvae are primarily in the ocean (Able et al. 2006; Witting et al. 1999). They have pelagic eggs and larvae (Fahay 1983; Witting et al. 1999). In contrast, Summer Flounder and Smallmouth Flounder spawn in the ocean, and larvae are either transported into the Bay or they enter as juveniles (Witting et al. 1999). Summer Flounder spawn from fall into winter as adults travel from inshore to offshore areas (Fahay 1983; Witting et al. 1999). Eggs and larvae are pelagic, and larvae are present in bays during fall–winter (Witting et al. 1999). Late juveniles and adult Summer Flounder are present in the Bay during summer and fall and are common predators of Winter Flounder young‐of‐the‐year (YOY) (Frisk et al. 2018; Taylor et al. 2019). Their occurrence overlaps with YOY Winter Flounder and Windowpane Flounder. Smallmouth Flounder spawn in the coastal ocean from summer to fall, with a peak in July–October (Fahay 1983). They have pelagic eggs and larvae, and larvae enter estuaries in the fall. Winter Flounder prey on YOY Smallmouth Flounder (Collette and Klein‐MacPhee 2002). For this group of species, therefore, temperature can shift migration patterns, spawning, and larval distributions in space and time, which in turn can influence predator–prey interactions, inducing shifts in the trophic dynamics. In addition, warming conditions can have complicated impacts on species. For example, the Black Sea Bass population has shifted to higher latitudes, but in these regions, the species has a shorter spawning season and lower gonadosomatic index, suggesting the potential for lower recruitment (Slesinger et al. 2021).

Curiously, even though local bottom temperature increased by 1.7°C during the study period (Figure S11), it was never selected as an important driver in any RDA analysis. Instead, AMO and lagged versions of AMO and NAO (AMO‐2 & NAO‐2) were selected. Time lags are a common feature of regime shifts since it takes time for the ecosystem to respond to environmental change (Báez and Real 2011; Beaugrand et al. 2015; Conversi et al. 2015). The correlation between bottom temperature and AMO was weak but significant (Spearman, *r* = 0.41, *p* = 0.015) and between NAO was nonsignificant (*r* = −0.06, *p* = 0.739). Removing all AMO and NAO variables in the RDA analysis resulted in selecting BT‐1, BT‐2, and chlorophyll *a* but the explained variance was almost half as when the climatic variables were included. Average annual bottom temperature, therefore, modulated the climatic drivers and was a less effective measure than AMO. This outcome also suggests that the processes affecting the community assemblage operated at a regional rather than local scale. Given the transient nature of the ecosystem, it is not surprising that regional drivers are important as they cue movement of migratory species that also tend to have top‐down effects on systems.

Secondarily, NAO was associated with community assemblage changes that were of shorter duration and occurred twice: 1987–1988 and 2009–2012. The negative phase of NAO, suppressing westerlies and otherwise impacting wind direction and intensity, the latitude of the Gulf Stream, mixed layer depth, and other factors (Taylor and Stephens 1998), was associated with high CPUE of Anchovies and probably Bluefish. Both Anchovies and Bluefish are coastal ocean spawners whose early life stages make extensive cross‐shelf migrations. Bluefish eggs and larvae are pelagic and larvae tend to occur between the surface and about 4 m depth (Shepherd and Packer 2006). Larval migration is facilitated by wind‐driven surface currents, warm‐core ring streams, and Gulf Stream filaments (Munch and Conover 2000; Hare et al. 2001). The pelagic juvenile stage makes an extensive cross‐shelf migration to enter estuaries. Anchovies spawn in the spring and summer, and they have pelagic eggs and larvae (Fahay 1983). Juveniles are found in estuaries in the summer but make winter migrations to the ocean (Munroe 2002). Adults occur in estuaries, the coastal ocean, and offshore to the Gulf Stream (Munroe 2002). Juveniles and adults are planktivores. A similar negative relationship between NAO and anchovies has been documented previously by Baez and Real (2011), who found that landings of the anchovy *Engraulis encrasicolus* in the Gulf of Cadiz were higher during a previous year negative NAO phase.

The 1999–2000 event and the 1987–1988 and 2009–2012 events appear to involve different drivers (AMO vs. NAO), different species, and likely different mechanisms. The climate drivers appear to act independently as the NAO‐induced 1987–1988 shift occurred during a cool AMO phase and the 2009–2012 shift during a warm AMO phase. All of these events fall within the definition of an “abrupt community/ecosystem shift” (ACS) as described by Beaugrand et al. (2008) and Beaugrand (2015). Their theory suggests that an ACS results from individual responses of species to a climate‐induced environmental change. This shift may not involve all species and may indirectly affect some species through trophic interactions. Beaugrand (2015) indicated that a step‐wise shift may be detected as a change along a PCA axis, as observed in the present study. Further Bourgrand (2015) regarded a regime shift as a special case of ACS.

Mollmann et al. (2015) in the same theme issue as Beaugrand et al. (2015) defined regime shifts as “dramatic, abrupt changes in the community structure that are persistent in time, encompassing multiple variables, and including key structural species—independently from the mechanisms causing them”. Under this definition, the 1999–2000 shift observed in this study qualifies as a regime shift. It was clearly “abrupt” and “persistent in time”, with the 1999–2000 shift prominent in both the K‐means and RDA analyses, identified in the change point analysis of the first RDA axis, and marking a separation in the finfish and mobile invertebrate community from a decade before to at least a decade after. The shift encompassed “multiple species” (at least 18) and included “key structural species” (e.g., complex trophic interactions between Winter Flounder, Windowpane Flounder, Summer Flounder, and Smallmouth Flounder as noted earlier). As far as being “dramatic”, it should be mentioned that in the RDA analysis, both AMO and the time delayed AMO‐2 were selected to explain changes along the 1st RDA axis. With both present, differential effects that depend on the relative weighting of the two variables on each individual species would distribute CPUE changes across multiple years, strongly suggesting that complex effects on age‐structure, migration, spawning, and species interactions were involved. Despite these species‐specific differential effects, the 1999–2000 event was the most distinct temporal shift in the data set and indicates the value of a long monitoring study in assessing community change.

The primarily NAO‐induced shifts of 1987–1988 and 2009–2012 fit the Mollmann et al. (2015) definition except for the “persistent in time” phrase. Although more limited in temporal extent and number of species involved, these NAO‐induced events are clearly climate driven. It is important to note that the Mollmann et al. (2015) definition does not include a requirement for alternative stable states as suggested in earlier investigations (e.g., Scheffer and van Nes 2004). The events in the present study strongly suggest that the persistence of the climate drivers maintain the community changes and that the community assemblage would not continue in the new structure without the forcing, as might be expected if alternative stable states were present.

Given the regional nature of the drivers associated with the observed community shifts, a similar pattern would be expected to be present in other local marine systems. This is in fact the case as reported by Howell and Auster (2012) for Long Island Sound and Collie et al. (2008) for Narragansett Bay. Using data from a trawl survey conducted in the Spring and Fall between 1984 and 2008, Howell and Auster (2012) reported a shift from “cold‐adapted” species to “warm‐adapted” species at about 1998–1999 and attributed it to increased bottom water temperatures. Their shift was identified by non‐metric multidimensional scaling, a multivariate ordination technique, and the community shift was distinctly evident only in the Spring samples. Eighteen of 49 species had significant trends at non‐Bonferroni corrected values of *p* < 0.01 in Spring and/or Fall based on the regression slope of the log mean CPUE vs. year. Several of the species identified as changing CPUE in the present study were identified in Howell and Auster (2012) as well. These included declines in Windowpane Flounder, Winter Flounder, and Fourbeard Rockling, and increases in Black Sea Bass, Scup, Smallmouth Flounder, Spotted Hake, and Summer Flounder. Howell and Auster (2012) did not include American Sand Lance or Anchovies in their study because they were undersampled in their trawl gear. The latter was the principal taxa driven by NAO‐induced changes in the present study. They also did not include invertebrates. Notably, a widespread die‐off of American lobsters occurred in 1999–2000 in Long Island Sound and the population has failed to recover (Lopez et al. 2013). Increased bottom water temperature has been implicated in this die‐off, and the timing suggests that this event could be related to AMO‐induced changes.

Collie et al. (2008) examined trawl survey samples collected from 1959 to 2005 at two locations in Narragansett Bay and found a progressive shift from vertebrates to invertebrates and from benthic to pelagic species. Analysis of temporal patterns in the community by non‐metric multidimensional scaling suggested clusters of years on a decadal scale. Nonparametric Mantel tests comparing matrices of single environmental variables to the community dissimilarity matrix found the strongest correlations with surface temperature and chlorophyll *a*, moderate correlations with NAO, and nonsignificant correlations with AMO. Surface temperature was used instead of bottom temperature in their study because of missing data in the bottom time series, and surface temperature, NAO, and AMO were lagged 0, 1, and 2 years in their analysis. These correlation results were opposite to the trend for the present study where the strongest relationship in RDA analysis of the temporal data was for AMO‐2, NAO‐2, and AMO. In the present study, bottom temperature and chlorophyll *a* were selected in the RDA analysis only when AMO and NAO variables were deleted, and the explained variance when using the local environmental variables was half that of climate variables. Since the Collie et al. (2008) time series was 47 years in length and ended in 2005, there would be little chance of detecting the regime shift that would have occurred in 1999–2000, since the post‐shift record was too short. Still, the 1990s and 2000–2005 years cluster as non‐overlapping groups in their ordinations for both sampling locations, so their results are consistent with the present study. In addition, Anchovies were not included in their analysis, so the NAO‐induced ACS of 1987–1988 and 2009–2012 observed in the present study would not have been detected by them.

Spatially, community structure variation was associated with the local factors bottom salinity (Sal), water depth (Depth), DO percent saturation (DOSat), the depth gradient during the trawl (delDepth), and Secchi depth (Secchi). The Peconic Estuary ecosystem is a west to east estuary with the largest surface freshwater source, the Peconic River, located to the west. Substantial freshwater also enters the system along the entire west–east extent through groundwater flow (Schubert 1998) and storm water runoff. Interestingly, while the K‐means analysis differentiated the eastern part of the study area from the rest of the system, there was no other west–east grouping of sampling stations based on community structure. Instead, the K‐means analysis differentiated between inshore and offshore locations outside of the eastern area. This probably reflects the substantial input of freshwater from runoff and groundwater flow from the land. This interpretation is consistent with the average salinity of the three regions with the highest salinity in the eastern region, intermediate salinity offshore in the western part of the system, and lowest salinities inshore (Figure S7).

The present study used a forward selection regression approach to identify the important local and regional environmental drivers of community structure in a large‐scale, temporally extensive monitoring data set. Comparison of this approach to Cloern et al. (2010) and Hughes et al. (2015) suggests there are several regression‐based approaches available to analyze large ecological data sets. Cloern et al. (2010) used two approaches to relate a fish and mobile invertebrate time series to the Pacific Decadal Oscillation (PDO) and the North Pacific Gyre Oscillation (NPGO). In the first, they created a community index from the first principal component (PC1) of an eighteen‐species fish and mobile invertebrate CPUE data set. They then developed an autoregressive model of order 1 to reconstruct PC1 with NPGO forcing. In the second, they related single species annual catch data to PDO and NPGO, individually or together. They found that inclusion of time lags optimized fits, and at least for several species, the most prominent change in catch occurred around 2000 linked to sign reversals in PDO and NPGO. Hughes et al. (2015) used sequential (i.e., forward selection) logistic regression to relate presence‐absence of two species of flatfish in shallow and deep nursery areas to local (temperature, salinity, sampling effort, dissolved oxygen [DO]) and regional (upwelling, El Nino Southern Oscillation [ENSO], and PDO) factors. Their analysis suggested that DO was the only consistent factor that explained flatfish presence in both nursery areas. They then used structural equation modeling to test hypothetical linear relationships between DO and potential forcing variables, finding that El Nino conditions and upwelling operated indirectly through changes in temperature, salinity, and precipitation to alter hypoxic conditions. Additionally, Hughes et al. (2015) used backward selection to relate flatfish recruitment and landings to one‐year lagged hypoxic conditions in the nursery area, upwelling, DO, PDO, NPGO, and ENSO. This analysis found that hypoxia in the nursery habitat was the strongest factor. A more detailed comparison of similarities and differences between these studies is included as Table S3. Examined together, these three studies highlight the value of extensive monitoring data sets and the importance of including regional forcing to fully quantify ecological relationships.

At first sight, the fraction of explained variation in the community structure obtained in the RDA analyses, ranging from 0.46 to 0.56 (Table 1), seems modest. These values are, however, comparable to those obtained by Flanagan et al. (2018) for RDA analyses of benthic fauna at five regional sites (0.42–0.52) using similar methods. In their study, Flanagan, et al. evaluated the amount of small‐scale heterogeneity in the benthic communities by fitting models to empirical variograms of the data. They found that 36%–59% of the total variation in the benthic communities represented small‐scale patchiness at a scale below the smallest sampling interval, and once adjusted for this patchiness, > 71% of the remaining variance was explained by the environmental variables selected in the RDA analysis. In the present study, this small‐scale patchiness represents intra‐annual and intra‐grid unit variability. While variogram models were not fit to the empirical variogram data in the present study, similar results would be expected, suggesting that the environmental variables chosen are explaining a substantial amount of the temporal and spatial community structure present in the Peconic Estuary system. Other indicators include the flatness of the residual variance in the variogram plots (Figures S5, S9, and S10), the fact that the confidence intervals for the empirical variograms do not include zero near the origin of the plots indicating substantial small‐scale heterogeneity, and the other diagnostic results reported. It should be noted that only when annual changes within all grid units were analyzed (34 years × 76 grid units), increasing variability in the data by two orders of magnitude, did the amount of explained variation fall below 40% in the RDA analyses.

The temporal and spatial patterns in community structure identified in the present study clearly frame the next steps that could be used to examine whether environmental changes have increased the vulnerability of individual finfish and mobile invertebrate species, community assemblages, and ecosystem properties. Information on the vulnerability of individual species and species assemblages can be obtained by examining how they alter their spatial distribution and habitat utilization in response to environmental change using techniques in Frisk et al. (2011). Linking spatial–temporal patterns to metrics derived from ecological relationships, such as life history characteristics (e.g., longevity, growth, and age at maturity), fishery status, level of exploitation, temperature tolerance, and migration patterns can add valuable information on vulnerability (Frisk et al. 2011). At the ecosystem level, Ecopath and its time and spatial varying companions, Ecosim and Ecospace, are quantitative modeling frameworks that represent all major ecosystem functional groups (Christensen and Pauly 1992; Pauly et al. 2000) and can be used to identify and assess structural changes in the ecosystem in response to environmental change (Odum 1971; Libralato et al. 2006; Christensen et al. 2009; Nuttall et al. 2011). They can be used to examine structural changes with particular attention to the temporal and spatial patterns identified. In addition, temporal changes in indices derived from cumulative biomass vs. trophic level and cumulative production vs. cumulative biomass curves can be used to assess periods of perturbation and recovery as proposed by Link et al. (2015).

Results of this study have several important conservation and management implications. First, abrupt community/ecosystem shifts (ACS), including a regime shift in 1999–2000, occurred in the Peconic Estuary community assemblage on a decadal time scale and these temporal changes were driven more by regional rather than local environmental drivers. Climate drivers causing abrupt changes were evident in the multivariate analysis, but local gradual or abrupt, natural or anthropogenic influences were not implicated in the temporal patterns for this ecosystem. No temporal trend or pattern was present in the RDA residuals after removing the effect of the climate drivers (Figure S5), suggesting that no major environmental driver was missed in the analysis. Second, regime shifts and other climate‐induced ACS events may not be accompanied by large, broad‐scale alterations of all parts in the ecosystem. The 1999–2000 regime shift, while clearly evident, involved about 18 of the 69 taxa examined. The trans periods (1987–1988 and 2009–2012) were marked by distinct changes in only those species that made large cross‐shelf migrations during early life stages. These results suggest that climate‐induced changes may require the analysis of extensive monitoring data to confidently detect and quantify their effect on the ecosystem.

Third, spatial habitats with different species assemblages were present (eastern, inshore, and offshore), but all three regions responded in a similar way in time to the climate drivers. These regions were characterized by differing Hellinger transformed CPUE in species such as Scup, Spider Crabs, and Black Sea Bass (eastern), Atlantic Silversides and Northern Pipefish (inshore), and Weakfish (offshore) (Figure 4). Not finding a strong west to east gradient in species assemblages was surprising, but the distinction between inshore and offshore regions is useful information for managers. For example, Suffolk County Department of Planning (2008) excluded areas within 1000 ft (305 m) of mean high water from development of shellfish aquaculture lease areas for various socio‐economic and environmental reasons. This restriction has additionally protected this distinct inshore fish and mobile invertebrate habitat. Given that the fauna analyzed are mobile, finding a similar regional community response in time to the climate drivers is a reasonable outcome. Since region‐specific assemblages are responding in a similar way (Figure 4), conservation and management decisions can be applied system‐wide and not restricted to specific geographic areas.

Finally, this study suggests that the response of the system to climate‐induced changes is predictable as long as extensive, up‐to‐date monitoring data remain available. Given that time lags are present in the system response, changes can be anticipated. Models along the line of the RDA analyses developed here, or more sophisticated and more general ecosystem models, are tools that can potentially be used effectively to scale the magnitude of the response to ACS events. Anticipating the occurrence of ACS events and estimating the scale of system response can lead to the development of more effective conservation and management strategies.

## Author Contributions

**Tyler R. Abruzzo:** conceptualization (equal), formal analysis (equal), investigation (equal), software (equal), validation (equal), visualization (equal), writing – original draft (equal), writing – review and editing (supporting). **Michael G. Frisk:** conceptualization (lead), funding acquisition (equal), investigation (equal), methodology (lead), supervision (equal), validation (equal), writing – original draft (equal), writing – review and editing (lead). **Liam Butler:** data curation (lead), formal analysis (equal), investigation (equal), methodology (equal), software (equal), validation (equal), visualization (equal), writing – original draft (equal), writing – review and editing (supporting). **Matthew Sclafani:** conceptualization (equal), investigation (supporting), validation (equal), writing – review and editing (supporting). **Paul Nunnenkamp:** data curation (lead), investigation (supporting), resources (lead), validation (equal), writing – review and editing (supporting). **Rachel Sysak:** data curation (equal), writing – review and editing (supporting). **Robert M. Cerrato:** conceptualization (lead), formal analysis (equal), funding acquisition (equal), investigation (equal), methodology (lead), project administration (lead), software (equal), supervision (equal), validation (equal), visualization (equal), writing – original draft (equal), writing – review and editing (lead).

## Conflicts of Interest

The authors declare no conflicts of interest.

## Supporting information


**Data S1.** Supporting Information.

## Data Availability

The Atlantic Multi‐Decadal Oscillation Index (AMO) and the North Atlantic Oscillation Index (NAO) were obtained from National Oceanic and Atmospheric Administration websites (psl.noaa.gov/data/timeseries/AMO/ and www.ncdc.noaa.gov/teleconnections/nao/). Chlorophyll data from a set of water quality monitoring stations was obtained from the Suffolk County Department of Health Services, Office of Ecology (Bureau of Marine Resources) at the following link gis.suffolkcountyny.gov/portal/home/item.html?id=5d4b53ec44204219a8da685f1859e096. Trawl data are posted at data.ny.gov/d/abww‐z5t5 and /d/9if6‐dz5v.
