# Supplementary material for: Coupled Multivariate Analyses Reveal Separate Climate and Local Drivers of Temporal and Spatial Change in a Coastal Marine Ecosystem
Source: Ecol Evol. 2025 Jun 27;15(7):e71637. doi: 10.1002/ece3.71637 (PMC12202972; doi:10.1002/ece3.71637)
Supplement: Supplementary file 1 — Data S1. Supporting Information. [file ECE3-15-e71637-s001.docx]

**Supporting Information**

**Coupled multivariate analyses reveal separate climate and local drivers of temporal and spatial change in a coastal marine ecosystem**

**Supplementary Text**

As with most ecological surveys, several taxa had very high catch per unit effort (CPUE) and frequency of occurrence in samples. Species with the highest CPUE included Scup (*Stenotomus chrysops*), Lady Crab (*Ovalipes ocellatus*), Anchovies (*Anchoa* spp), Weakfish (*Cynoscion regalis*), Winter Flounder (*Pseudopleuronectes americanus*), Herrings (*Clupeidae* spp, including *Clupea harengus*), Mantis Shrimp (*Squilla empusa*), Atlantic Silverside (*Menidia menidia*), Windowpane Flounder (*Scophthalmus aquosus*) in addition to a variety of species of Spider Crabs (*Libinia* spp) and Squid (*Loligo* spp) (Figure S1A). Species frequently caught in trawls included Lady Crab (73.0%), Scup (62.3%), Winter Flounder (55.5%), Anchovies (50.4%), Mantis Shrimp (39.5%), Summer Flounder (*Paralichthys dentatus*, 37.4%), Horseshoe Crab (*Limulus polyphemus*, 37.3%), Striped Searobin (*Prionotus evolans*, 37.0%), Weakfish (36.7%), and Windowpane Flounder (34.2%). A complete species list is provided as a supplementary table (Table S1).

Spearman’s rank correlation analysis for 69 species resulted in 19 taxa significantly (Bonferroni adjusted p <0.05) changing in rank abundance from 1987 to 2020 (Figure S1B). Some species, including Winter Flounder (r­_s_ = -0.831), Lady Crab (r­_s_ = -0.796), Horseshoe Crab (rs = -0.788), Windowpane Flounder (r_s_ = -0.779), Fourbeard Rockling (rs = -0.771), American Sand Lance (r_s_ = -0.659) and Bluefish (r_s_ = -0.564) showed a strong negative rank order correlation with year while Conger Eel (r_s_ = 0.565), Weakfish (r_s_ = 0.617), Atlantic Moonfish (r_s_ = 0.632), Spotted Hake (r_s_ = 0.676), Hogchocker (r_s_ = 0.729), Summer Flounder (r_s_ = 0.741), Scup (r_s_ = 0.754), Black Sea Bass (r_s_ = 0.804), Smallmouth Flounder (r_s_ = 0.806), Northern Kingfish (r_s_ = 0.835), Clearnose Skate (r_s_ = 0.839), and Bay Scallops (r_s_ = 0.858) showed a strong positive rank order correlation with year. Select environmental data are plotted in Figure S2.

Table S1. Common and scientific names of taxa in the study. Code is an eight character abbreviation of the common name used in RDA plots.

| **Common Name** | **Code** | **Species** |
| --- | --- | --- |
| American Eel | AmerEel | Anguilla rostrata |
| American Lobster | AmerLobs | Homarus americanus |
| American Sand Lance | AmerLanc | Ammodytes americanus |
| Anchovies | Anchspp | Anchoa spp |
| Atlantic Cod | AtlaCod | Gadus morhua |
| Atlantic Mackerel | AtlaMack | Scomber scombrus |
| Atlantic Menhaden | AtlaMenh | Brevoortia tyrannus |
| Atlantic Moonfish | AtlaMoon | Selene setapinnis |
| Atlantic Silverside | AtlaSilv | Menidia menidia |
| Atlantic Tomcod | AtlaTomc | Microgadus tomcod |
| Bay Scallop | BayScal | Argopecten irradians |
| Black Drum | BlacDrum | Pogonias cromis |
| Black Sea Bass | BlacBass | Centropristis striata |
| Blue Crab | BlueCrab | Callinectes sapidus |
| Blue Runner | BlueRunn | Caranx crysos |
| Bluefish | Bluefish | Pomatomus saltatrix |
| Butterfish | Buttrfsh | Peprilus triacanthus |
| Channeled Whelk | ChanWhel | Busycotypus canaliculatus |
| Clearnose Skate | CleaSkat | Raja eglanteria |
| Conger Eel | CongEel | Conger myriaster |
| Crevalle Jack | CrevJack | Caranx hippos |
| Cunner | Cunner | Tautogolabrus adspersus |
| Feather Blenny | FeatBlen | Hypsoblennius hentz |
| Flatclaw Hermit Crab | FlatCrab | Pagurus pollicaris |
| Fourbeard Rockling | FourRock | Enchelyopus cimbrius |
| Fourspine Stickleback | FourStic | Apeltes quadracus |
| Fourspot Flounder | FourFlou | Hippoglossina oblonga |
| Goby spp. | GobiGobi | Goby spp |
| Grubby | Grubby | Myoxocephalus aenaeus |
| Hard Clam | HardClam | Mercenaria mercenaria |
| Herrings (Clupeidae) | Herrspp | Clupea spp |
| Hogchocker | Hogchokr | Trinectes maculatus |
| Horseshoe Crab | HorsCrab | Limulus polyphemus |
| Inshore Lizardfish | InshLiza | Synodus foetens |
| Knobbed Whelk | KnobWhel | Busycon carica |
| Lady Crab | LadyCrab | Ovalipes ocellatus |
| Lined Seahorse | LineSeah | Hippocampus erectus |
| Little Skate | LittSkat | Leucoraja erinacea |
| Lookdown | Lookdown | Selene vomer |
| Mantis Shrimp | MantShri | Squilla empusa |
| Moon Snail | MoonSnai | Euspira heros |
| Naked Goby | NakeGoby | Gobiosoma bosc |
| Northern Kingfish | NortKing | Menticirrhus saxatilis |
| Northern Pipefish | NortPipe | Syngnathus fuscus |
| Northern Puffer | NortPuff | Sphoeroides maculatus |
| Northern Searobin | NortSear | Prionotus carolinus |
| Oyster Toadfish | OystToad | Opsanus tau |
| Pollock | Pollock | Melanogrammus aeglefinus |
| Red Hake | RedHake | Urophycis chuss |
| Rock Gunnel | RockGunn | Pholis gunnellus |
| Rough Scad | RougScad | Trachurus lathami |
| Scup | Scup | Stenotomus chrysops |
| Seaboard Goby | SeabGoby | Gobiosoma ginsburgi |
| Silver Hake | SilvHake | Merluccius bilinearis |
| Silver Perch | SilvPerc | Bairdiella chrysoura |
| Smallmouth Flounder | SmalFlou | Etropus microstomus |
| Smooth Dogfish | SmooDogf | Mustelus canis |
| Spider Crab | SpidCrab | Libinia spp. |
| Spot | Spot | Leiostomus xanthurus |
| Spotted Hake | SpotHake | Urophycis regia |
| Squid spp. | Squispp | Squid spp |
| Striped Searobin | StriSear | Prionotus evolans |
| Summer Flounder | SummFlou | Paralichthys dentatus |
| Tautog | BlacTaut | Tautoga onitis |
| Three-Spined Stickleback | ThreStic | Gasterosteus aculeatus |
| Weakfish | Weakfish | Cynoscion regalis |
| Windowpane Flounder | WindFlou | Scophthalmus aquosus |
| Winter Flounder | WintFlou | Pseudopleuronectes americanus |
| Winter Skate | WintSkat | Leucoraja ocellata |

Table S2. Spearman’s Rank Correlation (rho) and p-value between year and Hellinger transformed annual CPUE. Species in bold are significant at a Bonferroni adjusted 0.05 level for 69 species.

| **Species** | **rho** | **p-value** |
| --- | --- | --- |
| **WintFlou** | **-0.831** | **1.14E-09** |
| **LadyCrab** | **-0.796** | **1.88E-08** |
| **HorsCrab** | **-0.788** | **3.27E-08** |
| **WindFlou** | **-0.779** | **5.61E-08** |
| **FourRock** | **-0.771** | **9.51E-08** |
| **AmerLanc** | **-0.659** | **2.25E-05** |
| **Bluefish** | **-0.564** | **5.15E-04** |
| AtlaTomc | -0.512 | 1.97E-03 |
| AtlaSilv | -0.493 | 3.03E-03 |
| RedHake | -0.478 | 4.29E-03 |
| WintSkat | -0.445 | 8.35E-03 |
| OystToad | -0.417 | 1.43E-02 |
| Herrspp | -0.411 | 1.57E-02 |
| LittSkat | -0.388 | 2.34E-02 |
| CrevJack | -0.359 | 3.72E-02 |
| GrubScul | -0.348 | 4.39E-02 |
| AtlaMack | -0.302 | 8.25E-02 |
| SilvHake | -0.297 | 8.84E-02 |
| NortPipe | -0.286 | 1.02E-01 |
| RockGunn | -0.183 | 2.99E-01 |
| Anchspp | -0.183 | 3.01E-01 |
| NakeGoby | -0.164 | 3.55E-01 |
| RougScad | -0.094 | 5.98E-01 |
| SeabGoby | -0.094 | 5.99E-01 |
| FourStic | -0.087 | 6.23E-01 |
| ThreStic | -0.059 | 7.41E-01 |
| LineSeah | 0.013 | 9.40E-01 |
| Lookdown | 0.060 | 7.35E-01 |
| AmerLobs | 0.092 | 6.05E-01 |
| BlueRunn | 0.107 | 5.46E-01 |
| Pollock | 0.118 | 5.07E-01 |
| Buttrfsh | 0.207 | 2.39E-01 |
| Squispp | 0.208 | 2.38E-01 |
| NortPuff | 0.237 | 1.77E-01 |
| MantShri | 0.249 | 1.56E-01 |
| ChanWhel | 0.261 | 1.35E-01 |
| FourFlou | 0.266 | 1.28E-01 |
| InshLiza | 0.290 | 9.61E-02 |
| AtlaMenh | 0.325 | 6.07E-02 |
| AmerEel | 0.327 | 5.92E-02 |
| KnobWhel | 0.353 | 4.03E-02 |
| Cunner | 0.386 | 2.42E-02 |
| BlueCrab | 0.392 | 2.19E-02 |
| SilvPerc | 0.405 | 1.75E-02 |
| FlatCrab | 0.409 | 1.64E-02 |
| GobiGobi | 0.412 | 1.55E-02 |
| Spot | 0.430 | 1.11E-02 |
| BlacTaut | 0.449 | 7.68E-03 |
| NortSear | 0.464 | 5.68E-03 |
| SpidCrab | 0.472 | 4.85E-03 |
| StriSear | 0.479 | 4.19E-03 |
| HardQuoh | 0.489 | 3.33E-03 |
| AtlaCod | 0.505 | 2.31E-03 |
| MoonSnai | 0.512 | 1.95E-03 |
| FeatBlen | 0.515 | 1.85E-03 |
| SmooDogf | 0.520 | 1.62E-03 |
| BlacDrum | 0.543 | 9.06E-04 |
| **CongEel** | **0.565** | **4.93E-04** |
| **Weakfish** | **0.617** | **1.01E-04** |
| **AtlaMoon** | **0.633** | **5.94E-05** |
| **SpotHake** | **0.676** | **1.12E-05** |
| **Hogchokr** | **0.729** | **9.95E-07** |
| **SummFlou** | **0.741** | **5.52E-07** |
| **Scup** | **0.754** | **2.67E-07** |
| **BlacBass** | **0.804** | **9.95E-09** |
| **SmalFlou** | **0.806** | **8.84E-09** |
| **NortKing** | **0.835** | **7.92E-10** |
| **CleaSkat** | **0.839** | **5.68E-10** |
| **BayScal** | **0.858** | **9.28E-11** |

Table S3. Comparison of several regression-based analyses of large ecological monitoring studies.

|  | **Present Study** | **Cloern et al. 2010** | **Hughes et al. 2015** |
| --- | --- | --- | --- |
| **Environment** | Estuary | Estuary | Estuary and coastal |
| **Temporal Extent** | 1987-2020 | 1980-2008 | 1970-2010 |
| **Spatial Extent** | ~ 200 sq. km. | ~ 1,400 sq. km. | ~ 180 sq. km. |
| **Number of Sampling Sites** | 76 | 24 | 8 deep channel, 10 shallow margin, ~10 coastal |
| **Number of Taxa** | 69 fish and mobile invertebrates | 18 fish and mobile invertebrates | 2 flatfish |
| **Taxa Transformation** | Hellinger CPUE (Catch Per Unit Effort) | CPUE, log(Catch) | CPUE, presence/absence |
| **Local Environmental Variables** | Temperature, salinity, dissolved oxygen (DO), DO percent saturation, depth, Secchi depth, chlorophyll *a* | N/A | Temperature, salinity, DO, nitrate, precipitation, solar radiation, wind |
| **Regional Environmental Variables** | Atlantic Multidecadal Oscillation (AMO), North Atlantic Oscillation (NAO) | Pacific Decadal Oscillation (PDO), North Pacific Gyre Oscillation (NPGO) | Tl Niño Southern Oscillation (ENSO), PDO, NPGO, Monterey Bay upwelling |
| **Linear Models** | 1. Forward selection redundancy analysis (RDA) with all taxa | 1. Single species Generalized Linear Model (GLM) (Poisson family)  2. Autoregressive model of order 1 reconstructing first principal component of the taxa dataset | 1.Single species, forward selection GLM (binomial family)  2. Single species, backwards elimination multiple regression  3. Structural Equation Model (SEM) relating DO to regional and local drivers of hypoxia |
| **Model Time Unit** | Annual | Annual | Monthly, annual |
| **Variable Selection Criterion** | AIC (Akaike Information Criterion) | AIC | AIC |
| **Number of Selected Explanatory Variables** | 3-5 (temporal)  5 (spatial) | 2 | 1-5 |
| **Time Lags Identified** | 2 years (forward selection) | 2 years (fit optimization) | 1 year (cross correlation) |

**Supplementary Figure Captions**

Figure S1. K-Means cluster analysis of the spatially averaged temporal community structure data. Year groups are identified by color (left) and Calinski criterion vs. number of groups (right).

Figure S2. Change point analysis results of the annual community structure scores along the first RDA axis vs. year. Points are RDA scores, lines are randomly selected fits from the joint posterior, and the blue curve is the change point posterior density.

Figure S3. Observed Hellinger transformed annual CPUE (points) and RDA predicted values (lines) for the spatially averaged temporal analysis.

Figure S4. A) Hellinger transformed CPUE for the 20 species with highest average catch over the 34-year survey period. B) Spearman’s Rank Correlation between year and Hellinger transformed annual CPUE. Species’ bars above or below the horizontal blue lines are significant at a Bonferroni adjusted 0.05 level.

Figure S5. MSO variograms of Hellinger transformed annual CPUE data versus time interval. Solid lines delineate the Bonferroni-corrected point confidence envelope of the empirical variogram, open circles are the fitted (explained) variogram from RDA predictions, open squares are the residual variogram from RDA analysis, and crosses are the sum of the fitted and residual variograms. The empirical variogram is not shown for clarity. See the explanations of equations 1-2 for definitions of these quantities. Numbers above the interval axis are the number of sample pairs in each interval class. Solid squares, although absent in this plot would have indicated the presence of a significant autocorrelation in the residuals detected via Mantel tests. Sample pairs at intervals greater than half the maximum interval are grouped as one point to the right of the vertical dashed line. This grouped semivariance is not interpretable since some samples cannot be used in the variance calculation.

Figure S6. K-Means cluster analysis of the temporally averaged spatial community structure data. Grid-unit groups are identified by color (left) and Calinski criterion vs. number of groups (right).

Figure S7. Mean (+ standard errors) for eastern, inshore, and offshore environmental variables. Grid-unit groups chosen by K-means cluster analysis (Figure 3 and S6) and environmental variables selected by the RDA analysis (Figure 4).

Figure S8. Observed (x-axis) and RDA predicted (y-axis) Hellinger transformed annual CPUE for each spatial grid unit in the temporally averaged spatial analysis. Line is 1:1.

Figure S9. MSO variogram of the temporally averaged Hellinger transformed CPUE grid-unit data versus distance interval. The MSO variogram utilizes the RDA analysis result in Figure 3. See the Figure S4 caption for further information in interpreting elements of this figure.

Figure S10. MSO variograms of spatially averaged Hellinger transformed annual CPUE data versus time interval for the A) eastern, B) inshore, and C) offshore regional grid-unit groups identified by K-means cluster analysis (Figure S6). The MSO variograms utilize the RDA analyses in Figure 5. See the Figure S5 caption for further information in interpreting elements of this figure.

Figure S11. Annual mean values of select environment variables vs. year. Bottom temperature (A) and salinity (B) were calculated from trawl data. Annual AMO (C) and average winter NAO (January-March) (D) were obtained from National Oceanic and Atmospheric Administration sites (www.psl.noaa.gov/data/timeseries/AMO/ and [www.ncdc.noaa.gov/teleconnections/nao/](http://www.ncdc.noaa.gov/teleconnections/nao/)).


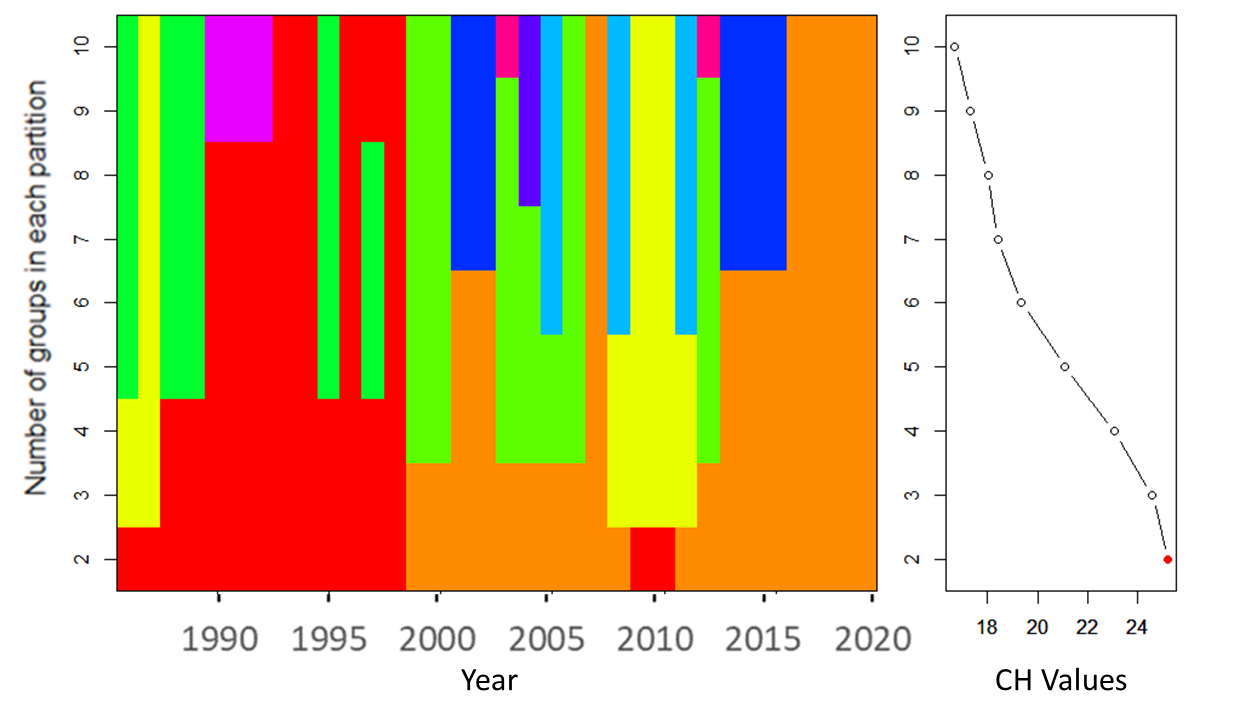
Figure S1

Figure S2


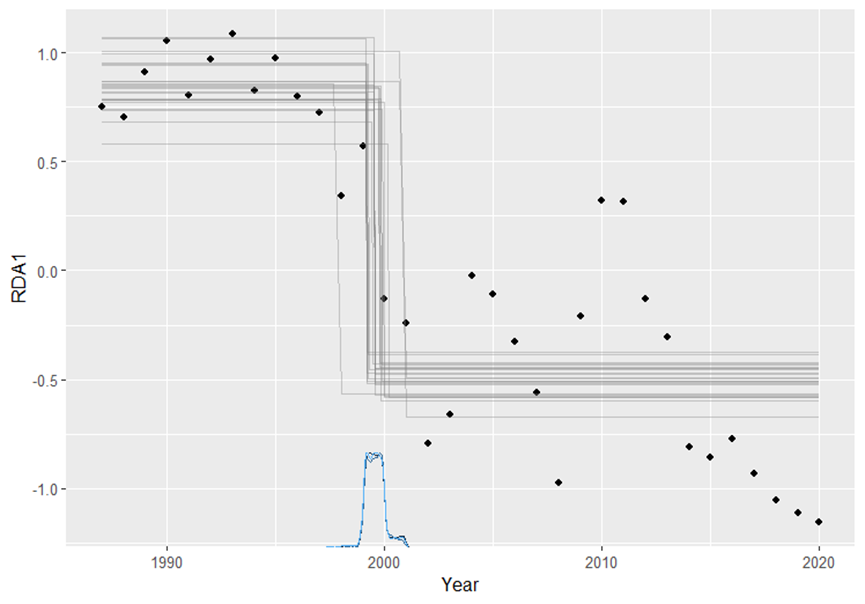


Figure
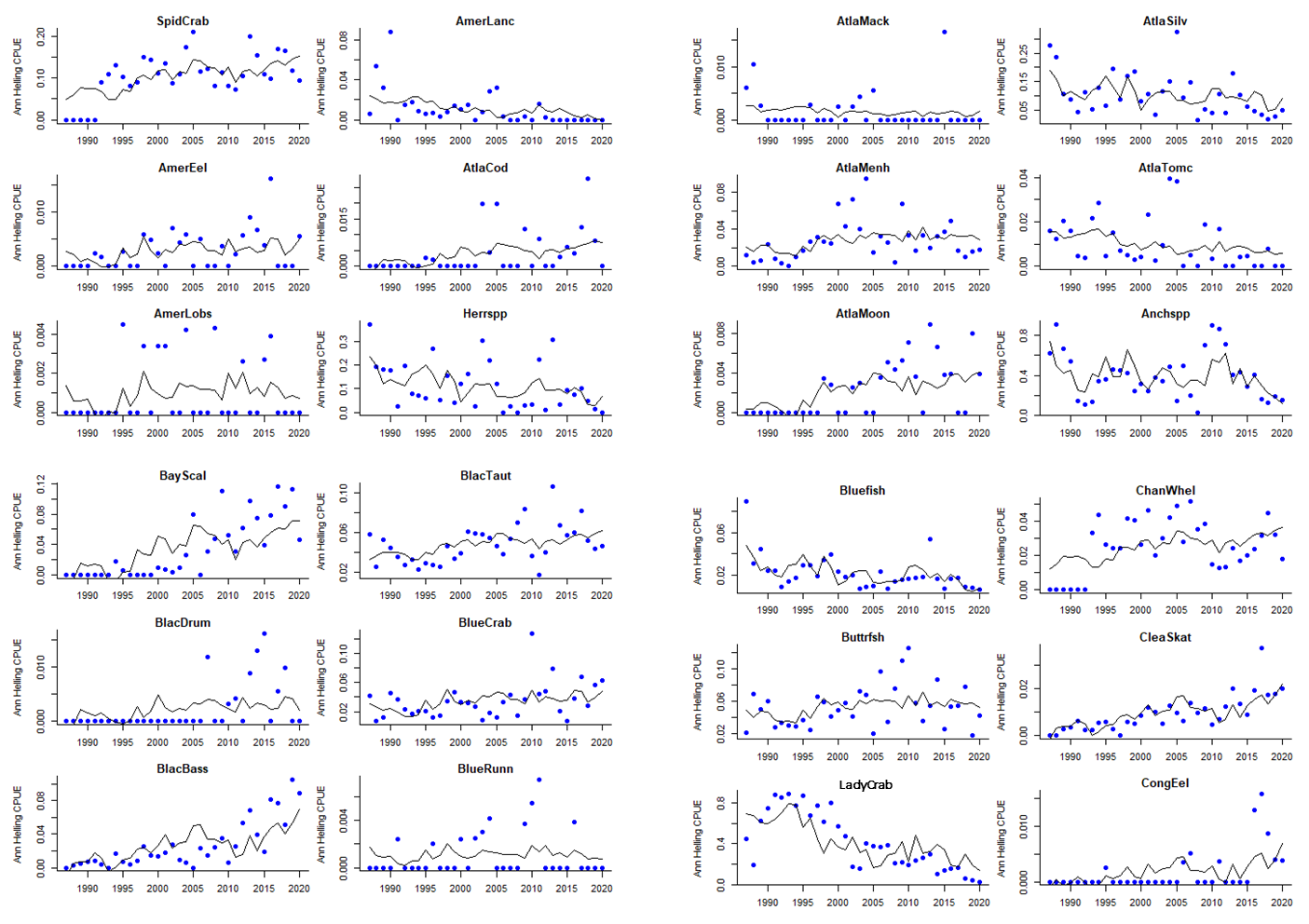
S3


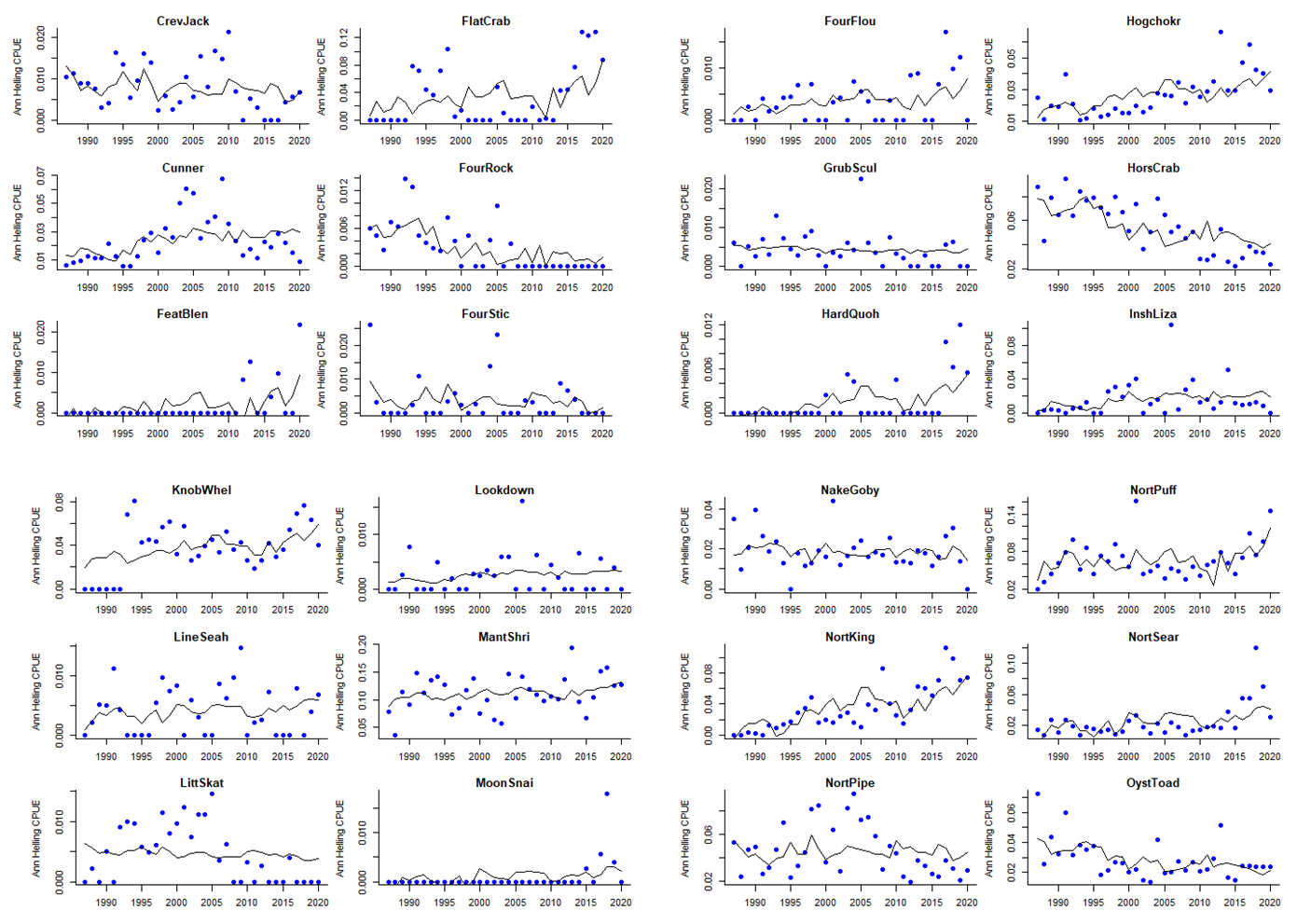

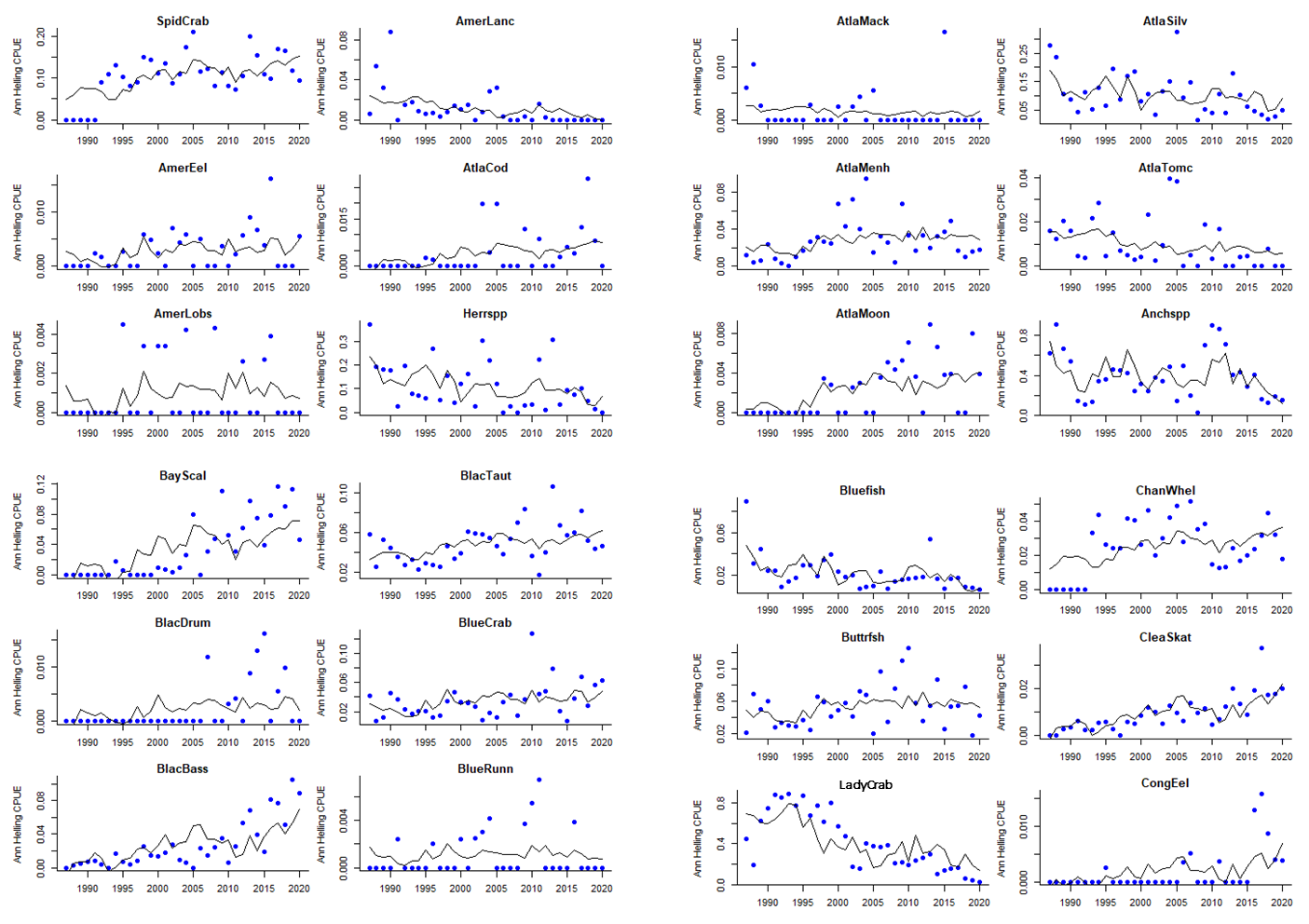
Figure S3 (contd)


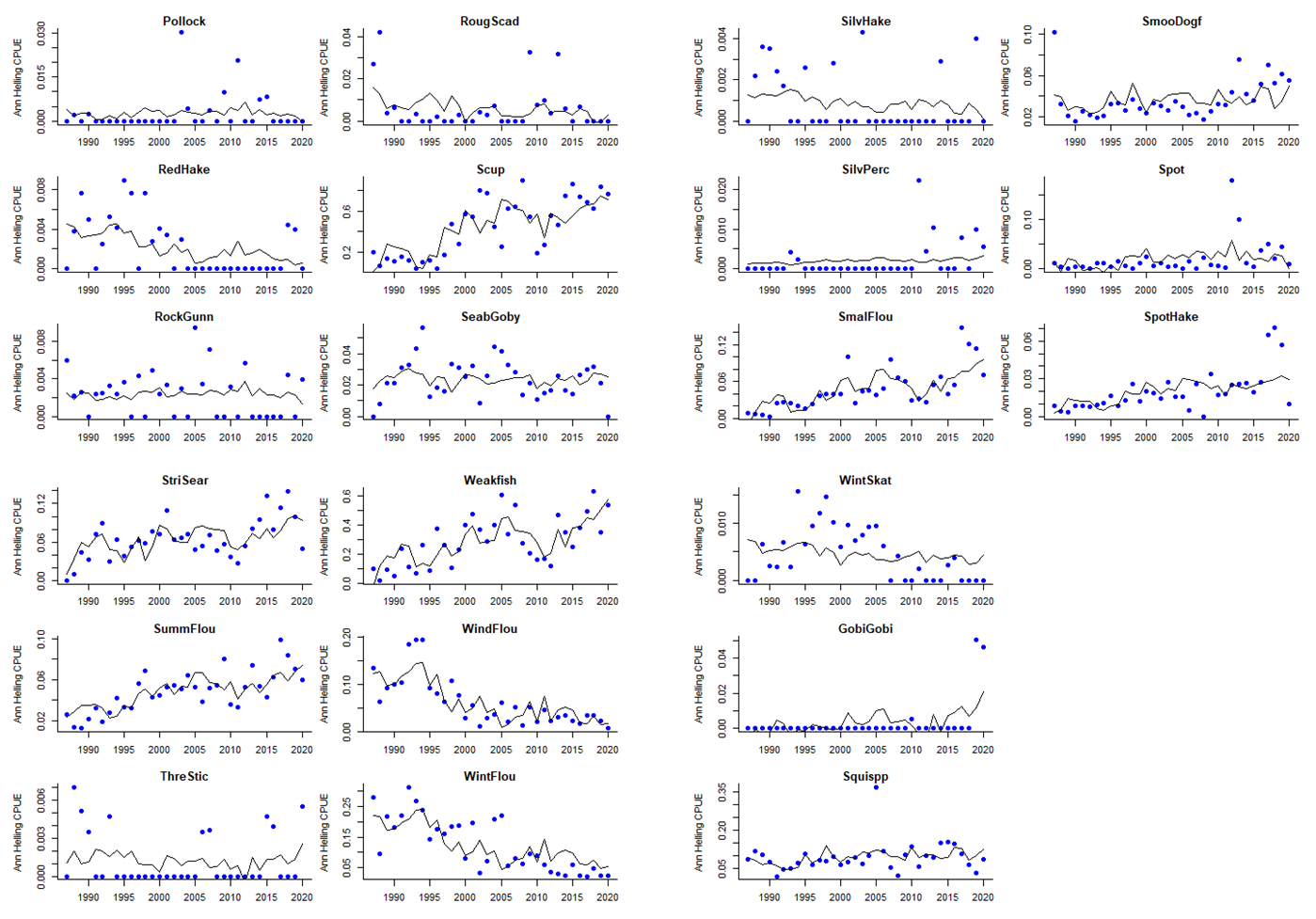
Figure S3 (contd)

Figure S4
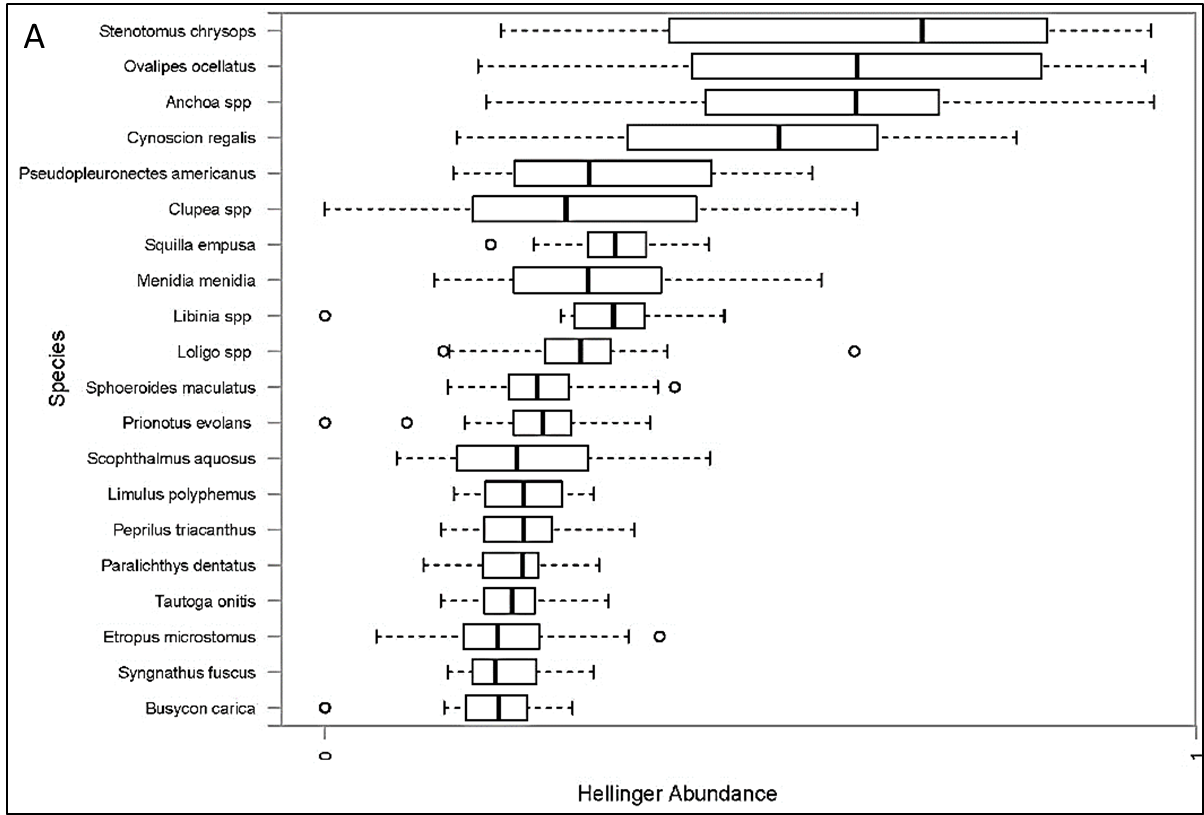


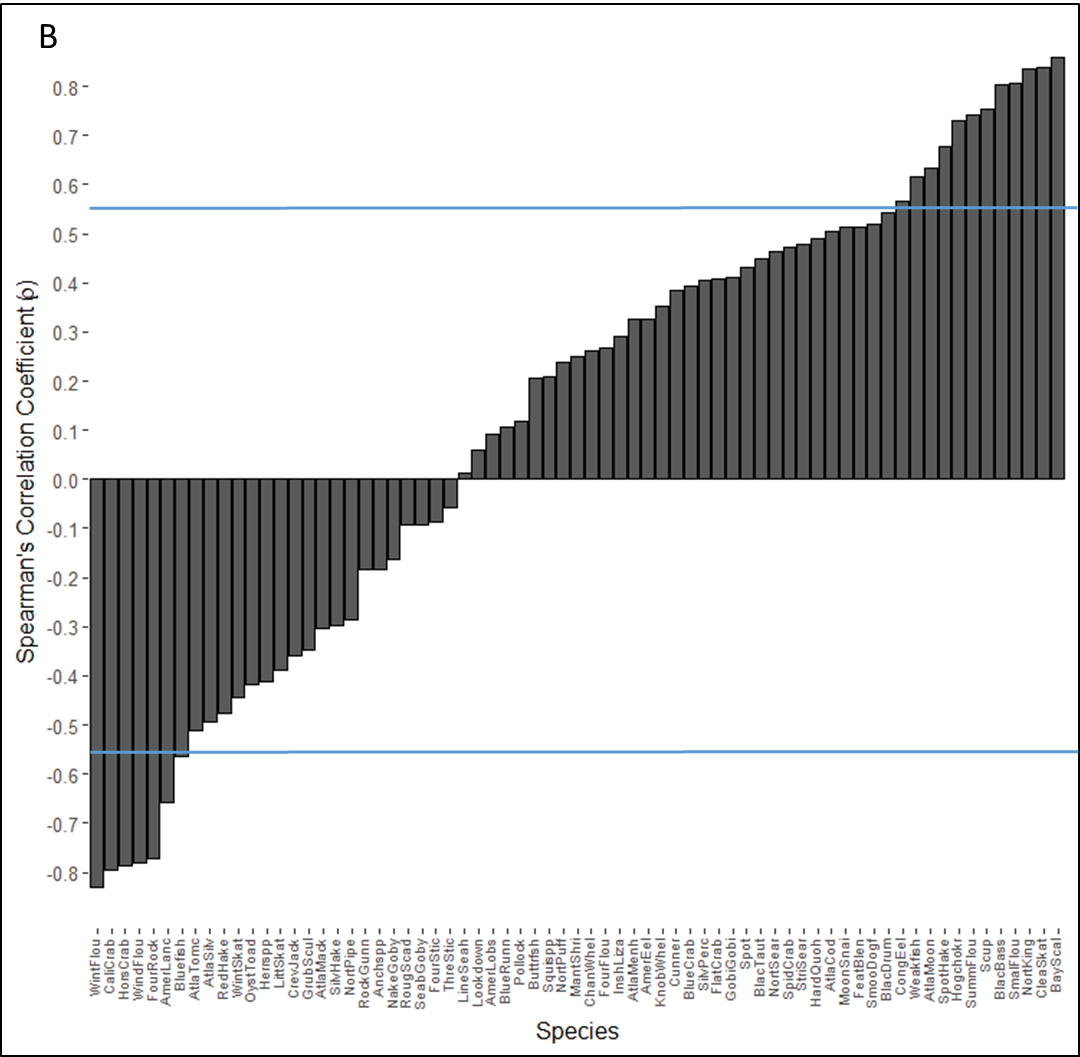
Figure S4 (contd)

Figure S5


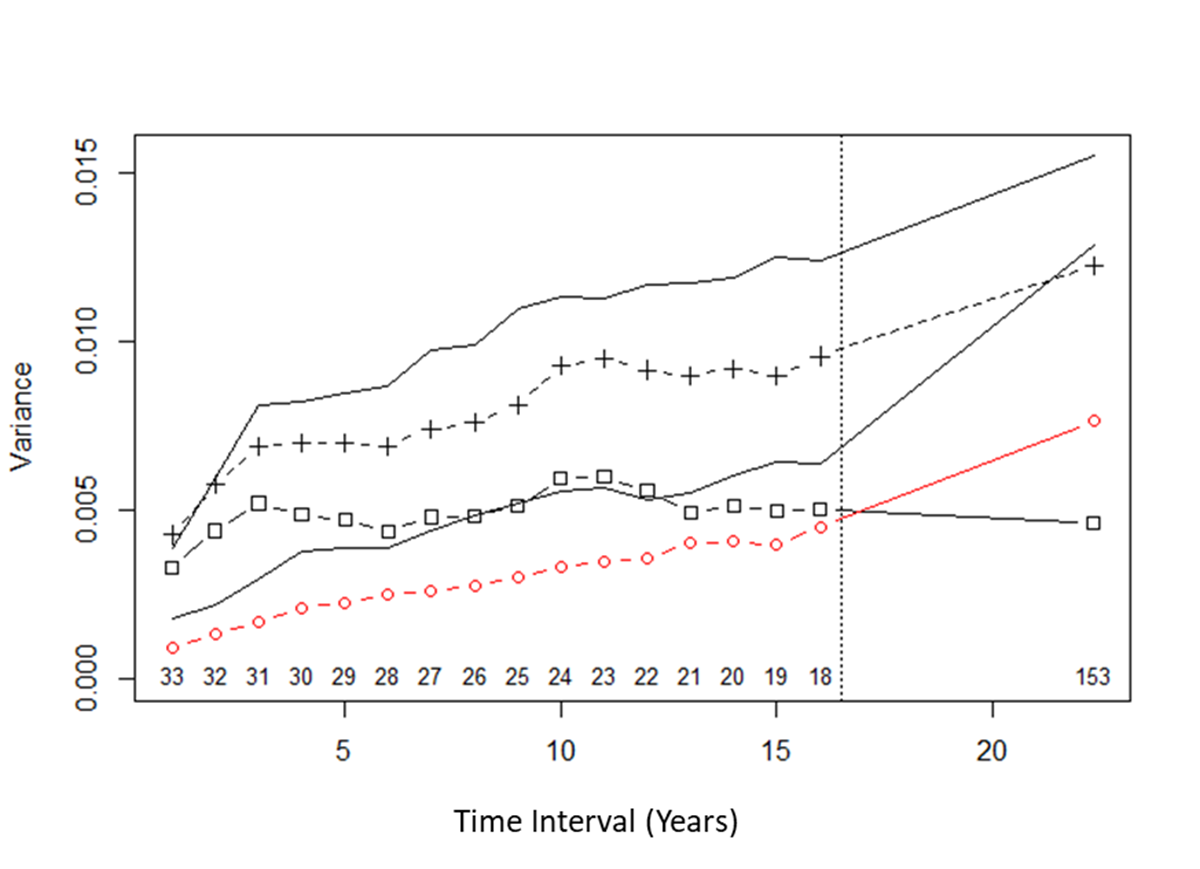


Figure S
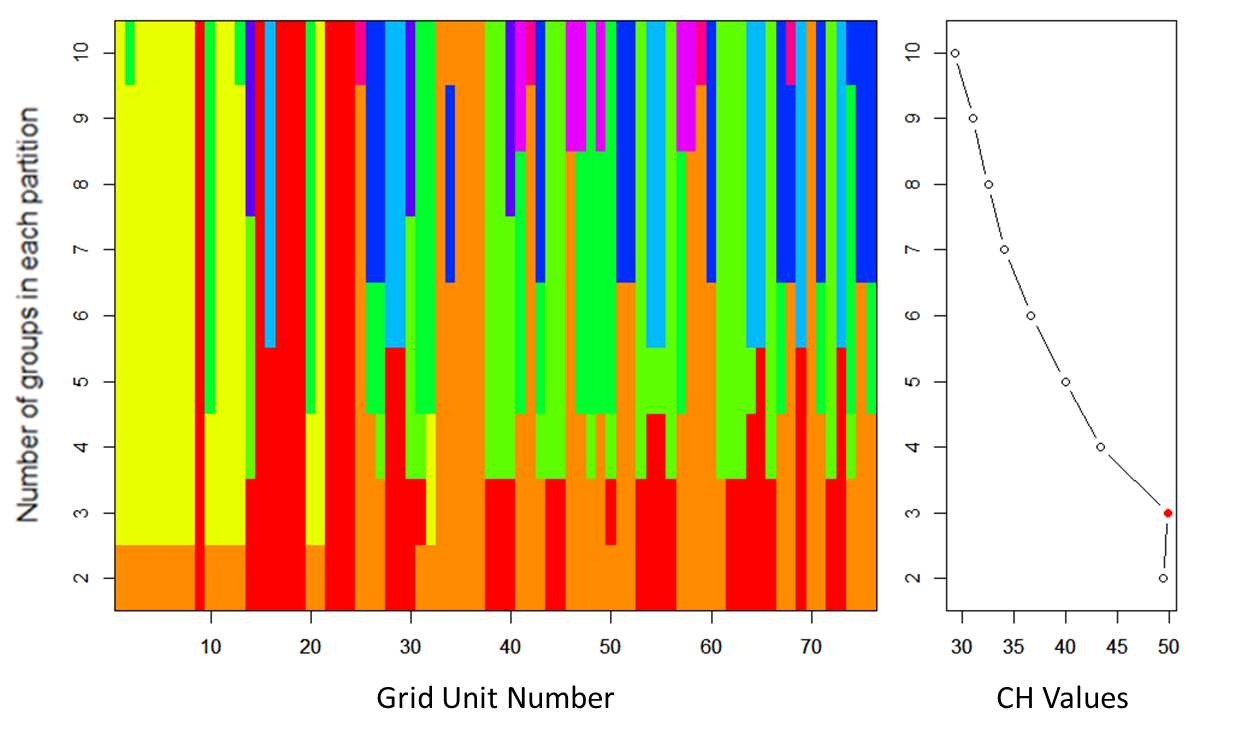
6

Figure S
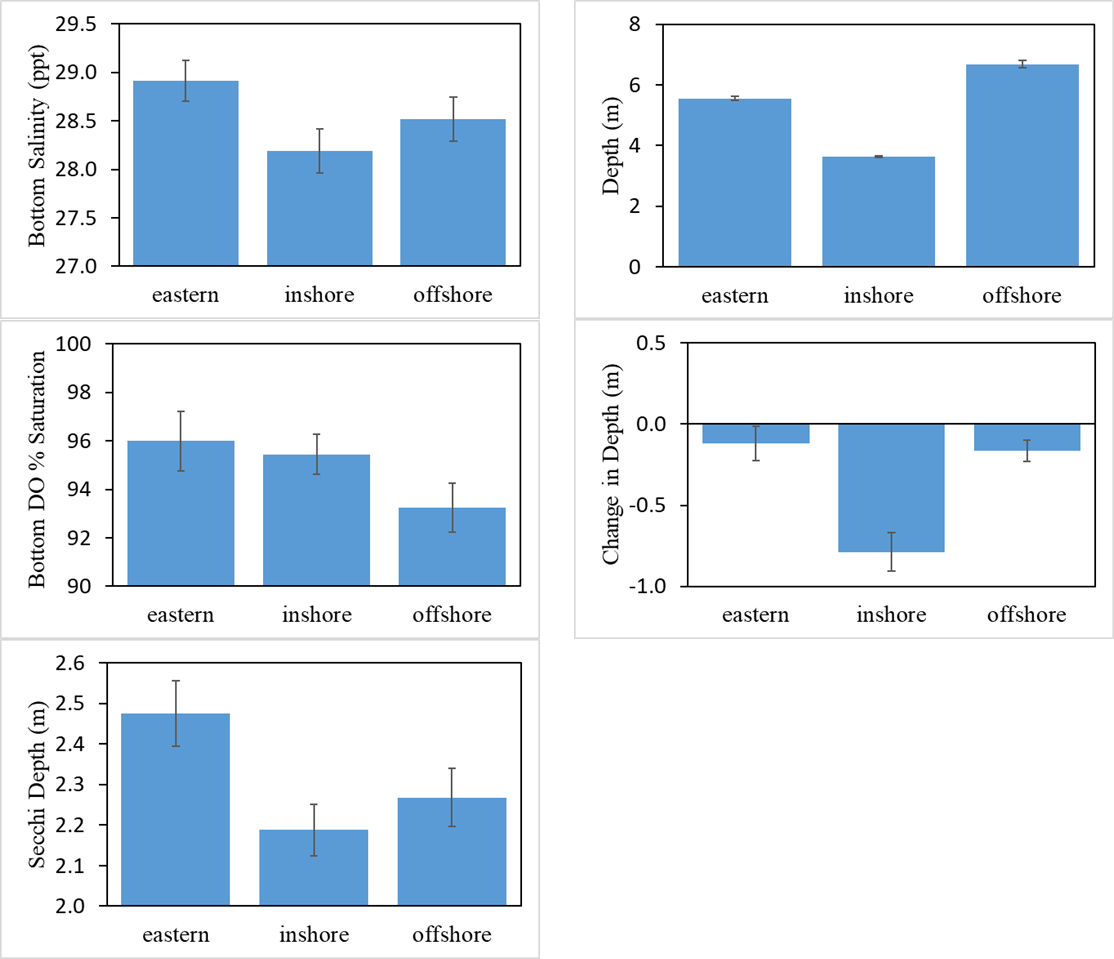
7


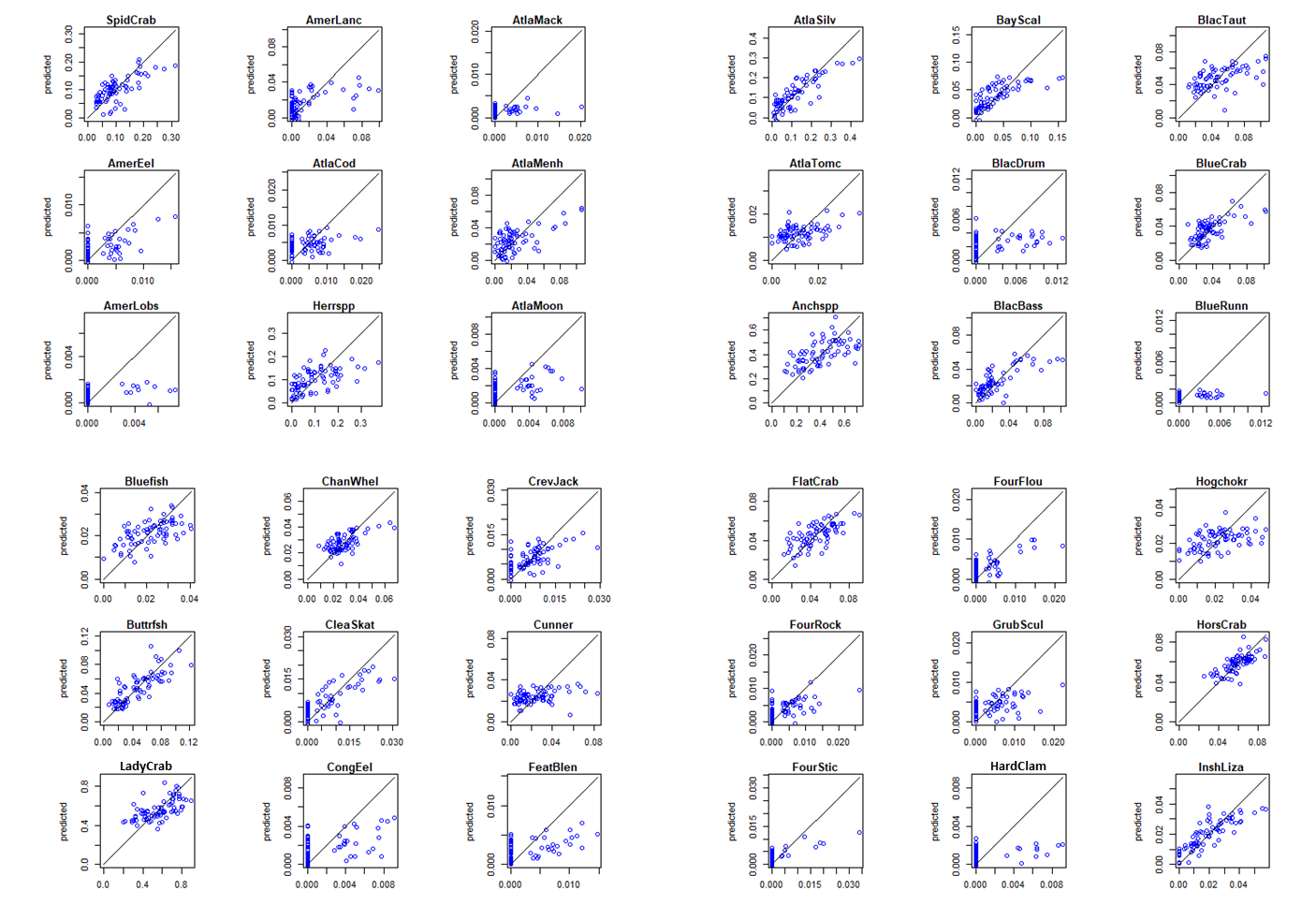
Figure S8


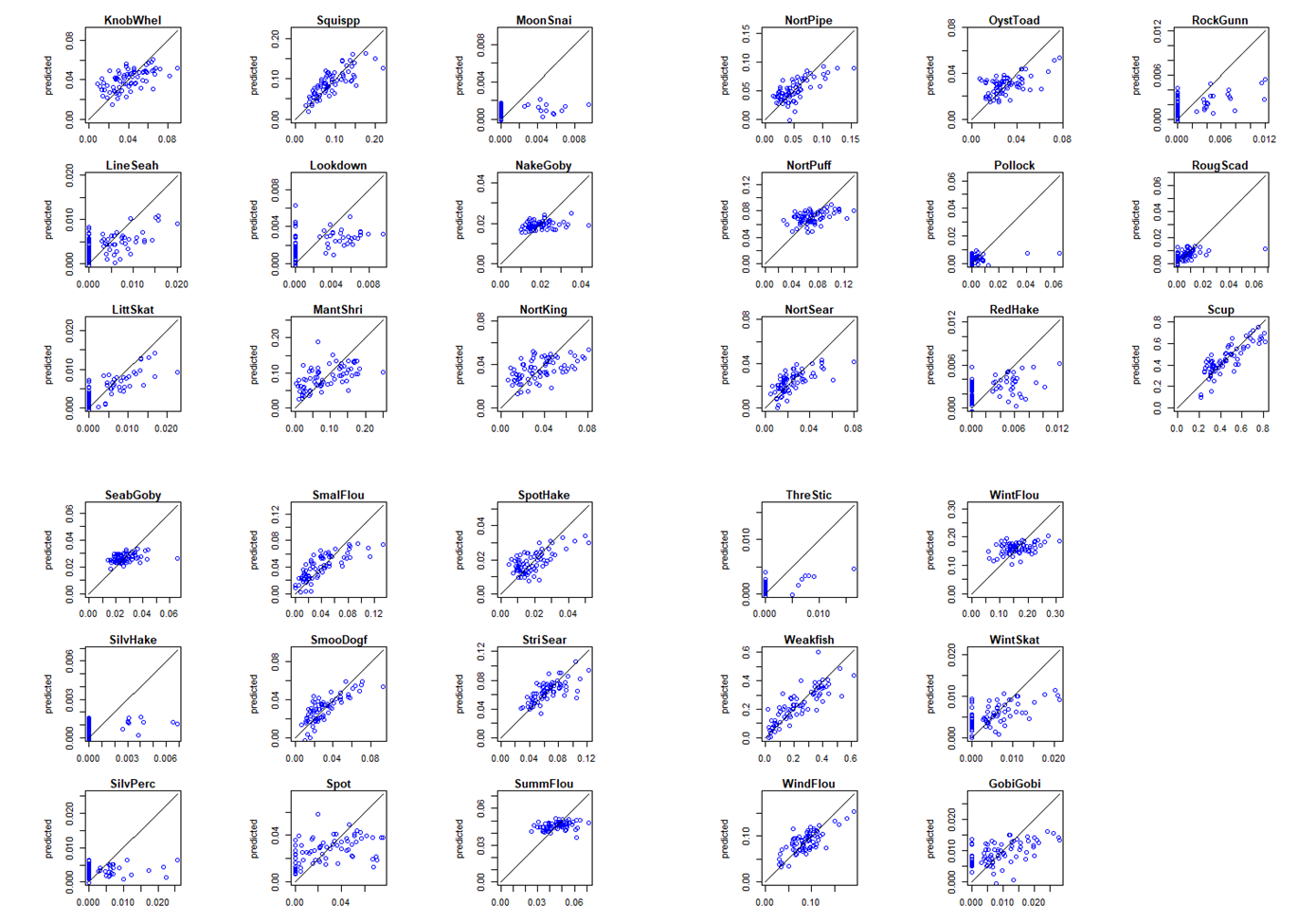
Figure S8 (Contd)

Figure S9


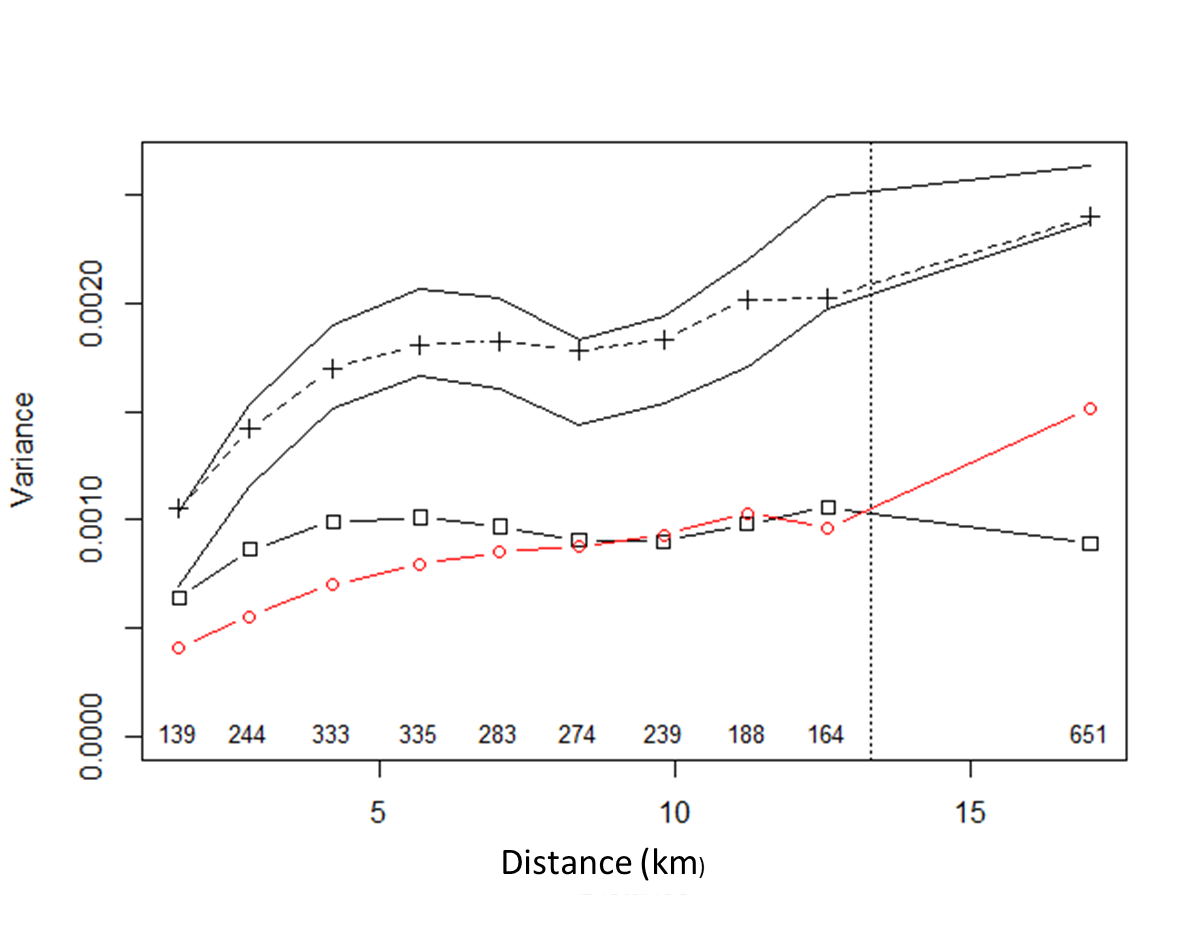


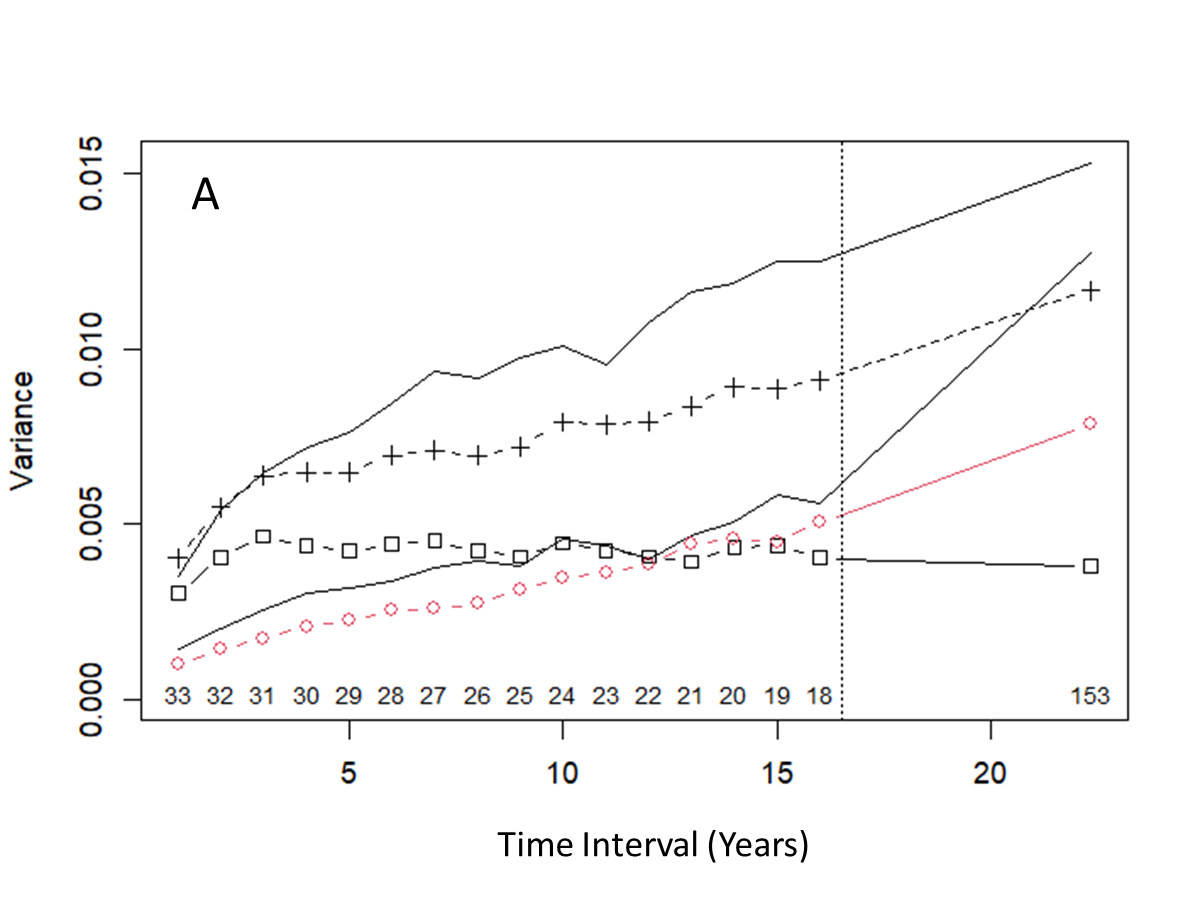
Figure S10

Figure S10 (contd)


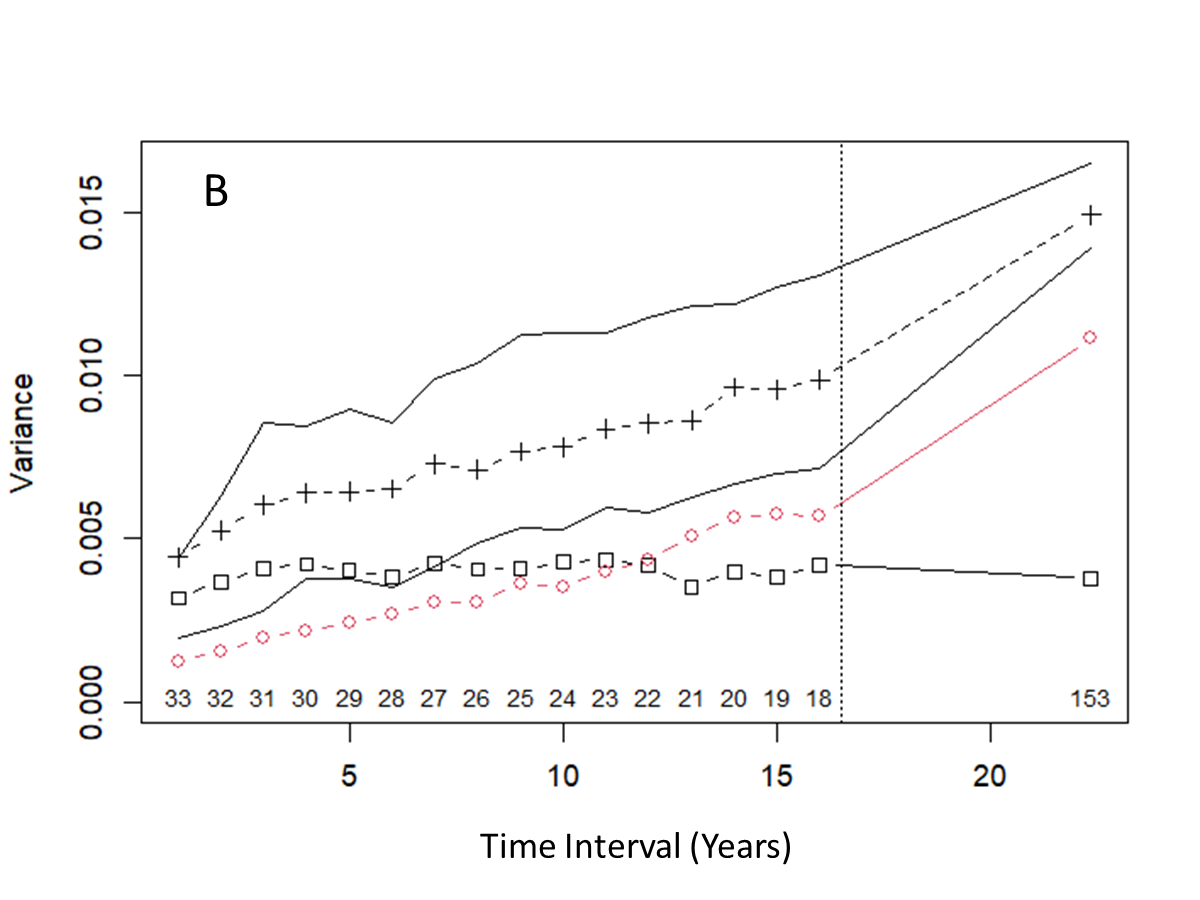


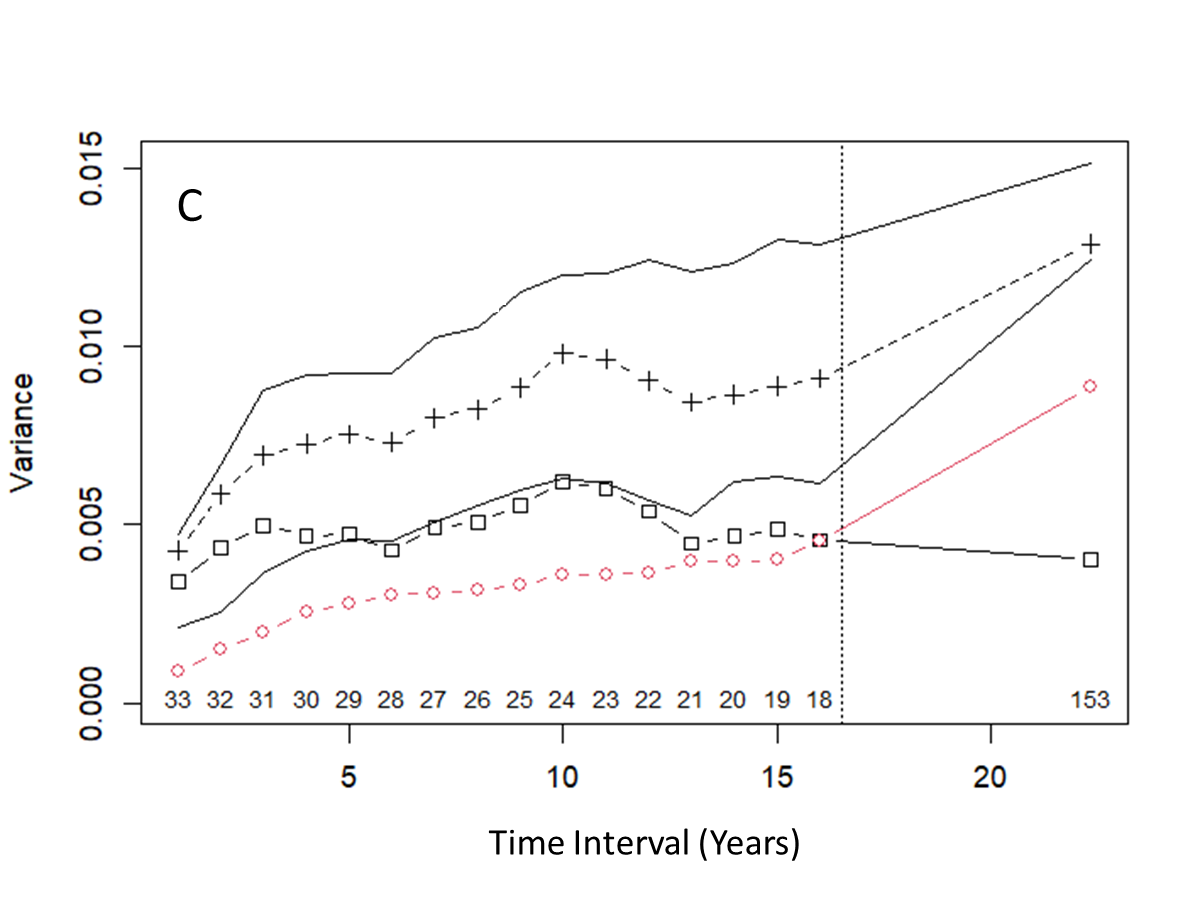
Figure S10 (contd)


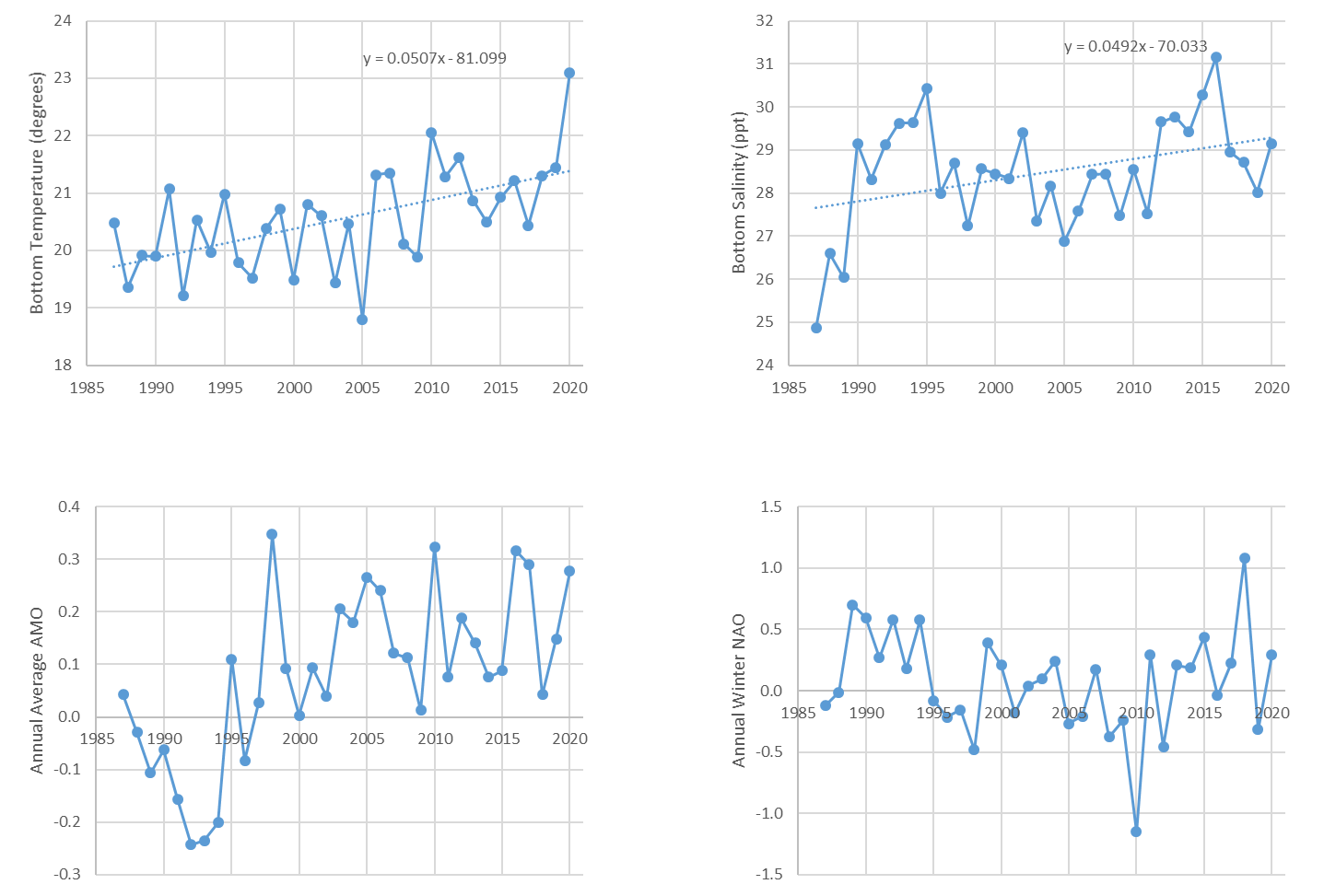
Figure S11

D

C

B

A
